# Identification and regulatory network analysis of SPL family transcription factors in *Populus euphratica* Oliv. heteromorphic leaves

**DOI:** 10.1038/s41598-022-06942-w

**Published:** 2022-02-21

**Authors:** Shao-Wei Qin, Liang-Hong Bao, Zhi-Gui He, Cai-Lin Li, Hong-gui La, Li-Feng Zhao

**Affiliations:** 1 School of Leisure and Health, Guilin Tourism University, Guilin, 541006 China; 2 Key Laboratory of Protection and Utilization of Biological Resources in Tarim Basin, Tarim University, Alar, 843300 China; 3 College of Life Sciences, Nanjing Agricultural University, Nanjing, 210095 China

**Keywords:** Developmental biology, Molecular biology, Plant sciences

## Abstract

The SQUAMOSA promoter-binding protein-like (SPL) family play a key role in guiding the switch of plant growth from juvenile to adult phases. *Populus euphratica* Oliv. exhibit typical heterophylly, and is therefore an ideal model for studying leaf shape development. To investigate the role and regulated networks of SPLs in the morphogenesis of *P. euphratica* heteromorphic leaves. In this study, 33 *P. euphratica* SPL (PeuSPL) genes were identified from *P. euphratica* genome and transcriptome data. Phylogenetic analysis depicted the classification of these SPL genes into two subgroups. The expression profiles and regulatory networks of *P. euphratica* SPL genes analysis displayed that major *P. euphratica* SPL family members gradually increases from linear to broad-ovate leaves, and they were involved in the morphogenesis regulation, stress response, transition from vegetative to reproductive growth, photoperiod, and photosynthesis etc. 14 circRNAs, and 33 lncRNAs can promote the expression of 12 of the *P. euphratica* SPLs by co-decoying miR156 in heteromorphic leaf morphogenesis. However, it was found that the effect of PeuSPL2-4 and PeuSPL9 in leaf shape development was contrasting to their homologous genes of *Arabidopsis*. Therefore, it was suggested that the SPL family were evolutionarily conserved for regulation growth, but were varies in different plant for regulation of the organ development.

## Introduction

*Populus euphratica* Oliv*.* is the only natural arbor tree species that grows in the desert area of northwestern China. Initially, the leaves of the *P. euphratica*are all linear (Li), with a leaf index (LI, leaf length/leaf width) ≥ 5 at the germination and seedling stages,. With increasing tree age, the leaves gradually became lanceolate (La, 5 > LI ≥ 2), ovate (Ov, 2 > LI ≥ 1), and broad-ovate (Bo, LI < 1) leaves^1,2^. Hence, *P. euphratica* is an ideal model to study leaf morphogenesis.

Transcription factors (TFs) are DNA-binding proteins in eukaryotes that specifically interact with cis-acting elements of certain gene promoter regions, and activate or inhibit gene transcription through specific interactions, thereby regulating the expression of downstream genes^3^. SBP1 and SBP2, which belong to the SQUAMOSA promoter-binding protein family (SPL), were discovered in *snapdragon*^4^. Subsequent studies showed that the SPL family plays a crucial role in the transition from juvenile to reproductive phase and are found in many plants, such as maize, tomato, alfalfa, and rice^5–7^. The SPL family can also affect leaf development^8^. For example, up-regulated SPL13 inhibits leaf primordia development in *Arabidopsis thaliana*, and delays the formation of the first true leaf^9^. SPL9 and SPL10 can change blade shape and promote epidermal hair formation on the distal axis of leaves^10^. The absence of SPL8 can lead to abnormal leaf development by preventing formation of normal leaf auricles and ligules^11,12^. Chen found that, in maize, SPL regulates plant epidermal cell differentiation and promotes epidermal hair formation on the abaxial surface of leaves to make the leaves exhibit adult characteristics by regulating miR172 expression^13^. Additionally, most SPLs could be regulated by miR156. For example, 11 SPLs have a miR156 response element in *Arabidopsis*. It indicates that miR156 can regulate SPL expression through cleavage or translational repression^14–16^.

In recent years, more studies on TFs in plants have been performed^17^, 28 full-length SPLs were identified in *Populus trichocarpa*^18^However, the role of SPLs in (*P. euphratica* heteromorphic leaves, *P.* hl) morphogenesis has remained unclear. In this study, SPL family expression profiles in Bo, Ov, La, and Li leaves of *P. euphratica* were analyzed by chain-specific sequencing technology. Combined with the competing endogenous RNA (ceRNA) hypothesis^19^, the networks of ceRNA (circRNA, lncRNA)-miRNA-SPL (mRNA) were constructed. Moreover, the roles of SPLs in *P*. hl morphogenesis were elucidated based on the above results.

## Materials and methods

### Plant materials, strand-specific sequencing, and miRNA sequencing

Growing young Bo, Ov, La, and Li leaves of *P. euphratica* were selected as experimental materials from the Tarim Basin, Xinjiang (81°17′56.52″ E, 40°32′36.90″ N). Sampling was done following standards for sampling and methods were conducted as described by Zhao and Qin^20^. All described methods were performed according to the relevant guidelines and regulations of China. Total RNA was extracted using the mirVanamiRNA Isolation Kit (Ambion) following the manufacturer’s protocol. The quality and concentration of isolated RNAs were evaluated by the Agilent Bioanalyzer 2100 (Agilent Technologies, Santa Clara, CA, USA). The strand-specific sequencing process and miRNA sequencing were performed as described by Levin and Qin, respectively^2,21^. Additionally, RNA sequencing was performed on the Illumina sequencing platform (OE Biotech, Shanghai, China).

### Identification of SPL TFs in *P. euphratica*

All candidate SPL genes were predicted from the *P. euphratica* genomic and transcriptome database^22,23^, and SPL amino acid sequences were obtained using BLASTP tool on NCBI (http://blast.ncbi.nlm.nih.gov/Blast.cgi). Homology alignment of the candidate SPL genes was carried out with the selected species. Only target sequences that met the criteria of sequence identity ≥ 80% and *e-*values < 10^−5^ were considered as the candidate SPL sequences that were homologous with the corresponding database sequences. SPL proteins transmembrane helix were predicted using TMHMM tools (http://www.cbs.dtu.dk/ services/TMHMM/).

### Properties analysis of SPL TFs in *P. euphratica*

Only sequences with full-length SBP domains were considered as SPL proteins for further analysis. The Hidden Markov model of the SPL domain was obtained from the Pfam database^24^. The SPL family database of other species was downloaded from the plant TFDB (http://planttfdb.gao-lab.org/)^25^. The properties of SPL proteins were analyzed using ExPasy web tools (https://www.expasy.org/tools). And motifs of SPL proteins were predicted with MEME tool (http://meme-suite.org/). Multiple sequence alignment of motif 1 and motif 2 of PeuSPLs were performed with COBALT tool (https://www.ncbi.nlm.nih.gov/tools/cobalt/).

### Phylogenetic analysis

Multiple sequence alignment and phylogenetic analysis of the amino acid sequence (full-length sequence or SPL domain sequence) of candidate SPL proteins were performed using the program MEGA7^26^ with default settings. The phylogenetic tree was constructed by the neighbor-joining method with 1000 bootstrap replicates. The concrete analysis method could be referred to Tamura^27^.

### RNA-seq data analysis

The transcriptome sequencing data were used to evaluate the expression profiles of miRNAs, lncRNAs, circRNAs, and mRNAs (including SPL genes and their target genes) in *P.* hl. Fragments per kb per million reads (FPKM)^28^ values were retrieved and normalized to estimate the expression level of lncRNAs and mRNAs. miRNAs were quantified and normalized to transcripts per million (TPM)^29^, and circRNAs were quantified as spliced reads per million reads (RPM)^30^. Heatmap was generated with help of a web tool from Omishare (https://www.omicshare.com/tools/Home/Soft/ heatmap). Additionally, fold changes in RNAs between two samples were calculated as log2 fold and data was normalized with DESeq2^31^.

### Interaction analysis between ceRNAs and SPL TFs, and network construction

According to the ceRNA hypothesis, psRNATarget (http://plantgrn.noble.org/psRNATarget/) was used to predict the target mRNAs, circRNAs, and lncRNAs of miRNA156. Then, differentially expressed lncRNA–mRNA and differentially expressed circRNA–mRNA pairs were identified based on the same miRNA response elements (MREs) and positive correlation of expression profiles, and both circRNA–miRNA–mRNA and lncRNA–miRNA–mRNA regulatory relationships were determined in La/Li, Ov/Li, Bo/Li, Ov/La, Bo/La, and Bo/Ov comparisons. Additionally, regulation network analysis was done using Cytoscape software^32^.

### Functional predictions of the SPL TFs

The SPL sequences of *P.* hl were compared with the *Arabidopsis* transcriptome (GCF_000001735.3_TAIR10_rna.fna.gz, https://www.ncbi.nlm.nih.gov/genome/?term=arabidopsis+thaliana) to obtain the homologous SPL genes of *Arabidopsis*. Ultimately, these homologous genes were analyzed by gene ontology (GO) enrichment. Furthermore, the relationships between SPL TFs were further elucidated using STRING network analysis (http://string-db.org/cgi/input.pl?sessionId=P30BEJCLYTAP&input_page_show_search=on).

### qPCR validation

Quantitative real-time polymerase chain reaction (qPCR) was carried out to verify the expression of circRNAs, miRNAs, and mRNAs. For mRNAs, lncRNAs, and circRNAs, the total RNA was extracted from the collected leaves of *P. euphratica* (Li, La, Ov, and Bo) using Trizol reagent (Invitrogen, Carlsbad, CA, USA) and treated with DNase I (Takara, Dalian, China) according to the manufacturer's protocol. For mRNA and circRNA, the purified RNA was subjected to reverse transcription to obtain cDNA using the PrimeScript II 1st Strand cDNA Synthesis Kit (Takara) with 1 μg total RNA and random 6-mers; alternatively, lncRNAs were reverse-transcribed using lnRcute lncRNA 1st Strand cDNA Synthesis Kit (Tiangen, Beijing, China) with 1 μg total RNA and random 6-mers. A Takara kit was used for circRNA and mRNA detection, a lnRcute lncRNA qPCR Kit (SYBR Green) (Tiangen, Beijing, China) was used for lncRNA detection, and 18S RNA was used as the internal reference. Primers were designed and provided by Sangon Biotech (Shanghai) Co., Ltd (Shanghai, China). The primer sequences are displayed in Table S1. Quantification of RNAs expression (circRNAs, lncNAs, and mRNAs) was performed using the comparative Ct method. The expression levels of RNAs were normalized as the ratio with 18S. Three biological replicates and three technical replicates were used for each RNA sample. The experiment was carried out according to the method described by Bao^33^.

## Results

### Identification of SPL TFs in *P.* hl

A total of 78 members of the SPL family were identified from the *P. euphratica* genome and transcriptome. Among them, 45 were identified as redundant sequences that were a product of alternative splicing and thus discarded (Table S2), and the Hidden Markov models of the remaining SPL proteins were determined using the Pfam database. All of these SPL proteins contained a conserved SBP domain (pfam03110) (Table S2). ExPasy was used to predict the physical and chemical properties of SPL TFs in *P. euphratica*, and found the length of the peptide chain of these SPL TFs ranged from 138 to 1073 amino acids, the molecular weight (Mw/Da) lies within the range of 15,225.5 to 118,988.54 Da in *P. euphratica* (Table S3). In addition, the theoretical isoelectric point of most SPL proteins in *P. euphratica* were slightly alkalescent (7.99–9.49), and only nine SPL proteins were acidic (6.02–6.99) (Table S2). The majority of SPL proteins contains fewer negative amino acids (Asp and Glu) than positive amino acids (Arg and Lys) in *P. euphratica* (Table S3). The average hydropathy of all SPL proteins varied from − 1.34 and − 0.27, which indicated that these proteins are hydrophilic (Table S3). The instability index of most PeuSPL proteins exceeded 40 (42.63–86.65), which indicated that these proteins are unstable (Table S3).

### Phylogenetic analysis of SPL proteins

**Figure 1:**
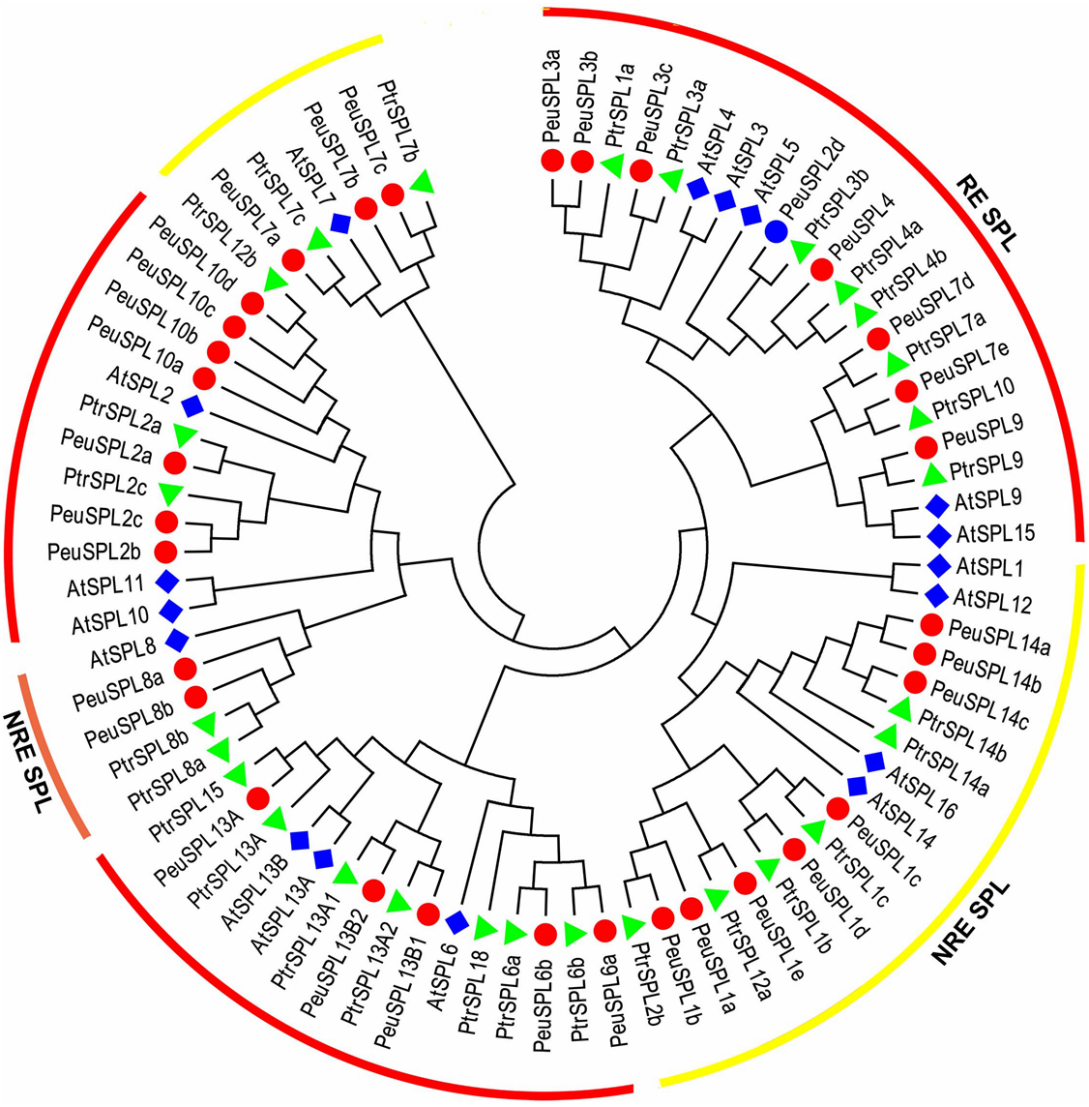
Phylogenetic analysis of SPL proteins in *P. euphratica*, *P. trichocarpa*, and *Arabidopsis*. The phylogenetic tree was built based on multiple sequence alignments of the SBP domain in the SPL proteins using the neighbor-joining method with 1000 bootstrap replicates. The red circles represent *P. euphratica*, and the diamonds and triangles represent *Arabidopsis* and *P. trichocarpa*, respectively*.* This figure was generated by MEGA 7 (https://www.megasoftware.net/).

A phylogenetic analysis of SPL TFs from *P. euphratica*, *Arabidopsis*, and *P. trichocarpa* was conducted to clarify the evolutionary relationship. We obtained 68 SPL TFs in *P. trichocarpa* and 30 in *Arabidopsis* from the Plant Transcription Factor Database Version 5 (PlnTFDB; http://planttfdb.gao-lab.org/download.php). Among these TFs, 52 were identified as redundant sequences from alternative splicing and thus discarded. The phylogenetic tree was constructed with the alignments of the remaining SPL TFs, which included 33 from *P. euphratica*, 28 from *P. trichocarpa*, and 17 from *Arabidopsis*, by the neighbor-joining method (Fig. 1). The SPL TFs of *P. trichocarpa* were assigned names such as PtrSPL1a and PtrSPL1b; *Arabidopsis* was treated in the same manner, as were the 33 SPL TFs in *P. euphratica* based on their homology with AtSPLs (*e.g.*, PeuSPL1a and PeuSPL1b). Based on homology with *Arabidopsis* and their genomic structural characteristics, 78 SPL proteins were divided into two subgroups: class I (yellow) and class II (red and light red) (Fig. 1). Class II was further subdivided into two branches: SPL proteins with (red) and without (light red) miR156 response elements (Fig. 1). According to the phylogenetic tree, the distribution of SPL proteins was uneven; there were only 25 proteins in class I, and the others were classified in class II proteins (Fig. 1).

### Conservative domain analysis of SPL proteins

**Figure 2:**
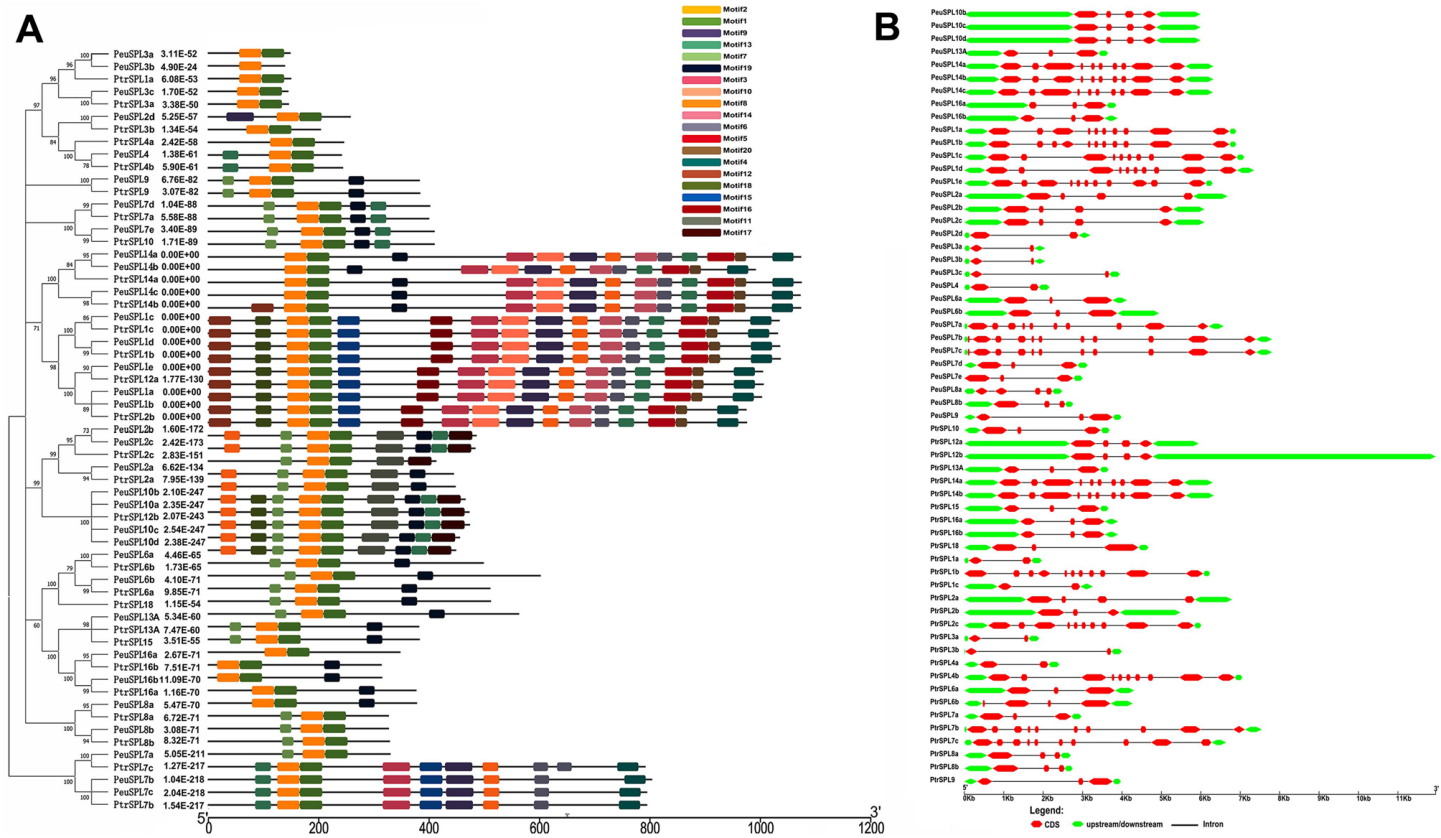
Genomic structure and motif composition of SPL proteins. (**A**) Motif analysis of SPL proteins from *P. euphratica* and *P. trichocarpa*. and conserved motifs in the SPL proteins are represented by colored boxes. The phylogenetic tree was constructed by MEGA 7 (https://www.megasoftware.net/), motifs in the SPL proteins were elucidated by MEME 5.0.1(http://meme-suite.org/), (**B**) Genomic structure of poplar SPL genes. Exons, introns, and UTRs are shown with red boxes, green boxes and black lines, respectively. Genomic structure was constructed by TBtools (https://www.tbtools.com/).

To further analyze the SPL sequence characteristics in *P.* hl, a comparative analysis of the conserved motifs was performed between *P. euphratica* and *P. trichocarpa* proteins. Twenty motifs (motifs 1–20) were predicted to reveal SPL protein structure details using MEME 5.0.1 (Fig. 2A). The information of each identified motif is shown in Figure S1. Among the 20 motifs, motif 1 and motif 2 both were identified as the conserved SPL domain, whereas no matches were found for the other motifs. Either motif 1 or motif 2 were nearly present in each PtrSPL and PeuSPL protein (except PeuSPL3b, which had no motif 1) (Figure S2), which provided further support for the reliability of identification, and indicated that these two motifs might play an important role in the SPL family. In general, SPL proteins clustered in the same subgroups shared similar motif compositions (Fig. 2A), which supports that there is functional similarities among members of the same subgroup. The differences in motif distribution among the subgroups of SPL genes revealed that the functions of these genes may have diverged during evolution. Furthermore, most of these SPL genes (22/61) contained three exons and two introns, 11 SPL genes possessed four exons and three introns, and 10 SPL genes comprised two exons and one intron, whereas 18 genes consisted of more than nine introns and 10 exons, of which five SPL genes contained 11 exons (Fig. 2B). Moreover, similar exon/intron structures were found in the same phylogenetic subgroup, which further confirmed the reliability of phylogenetic analysis.

### Expression profiles of SPL genes in *P*. hl

**Figure 3:**
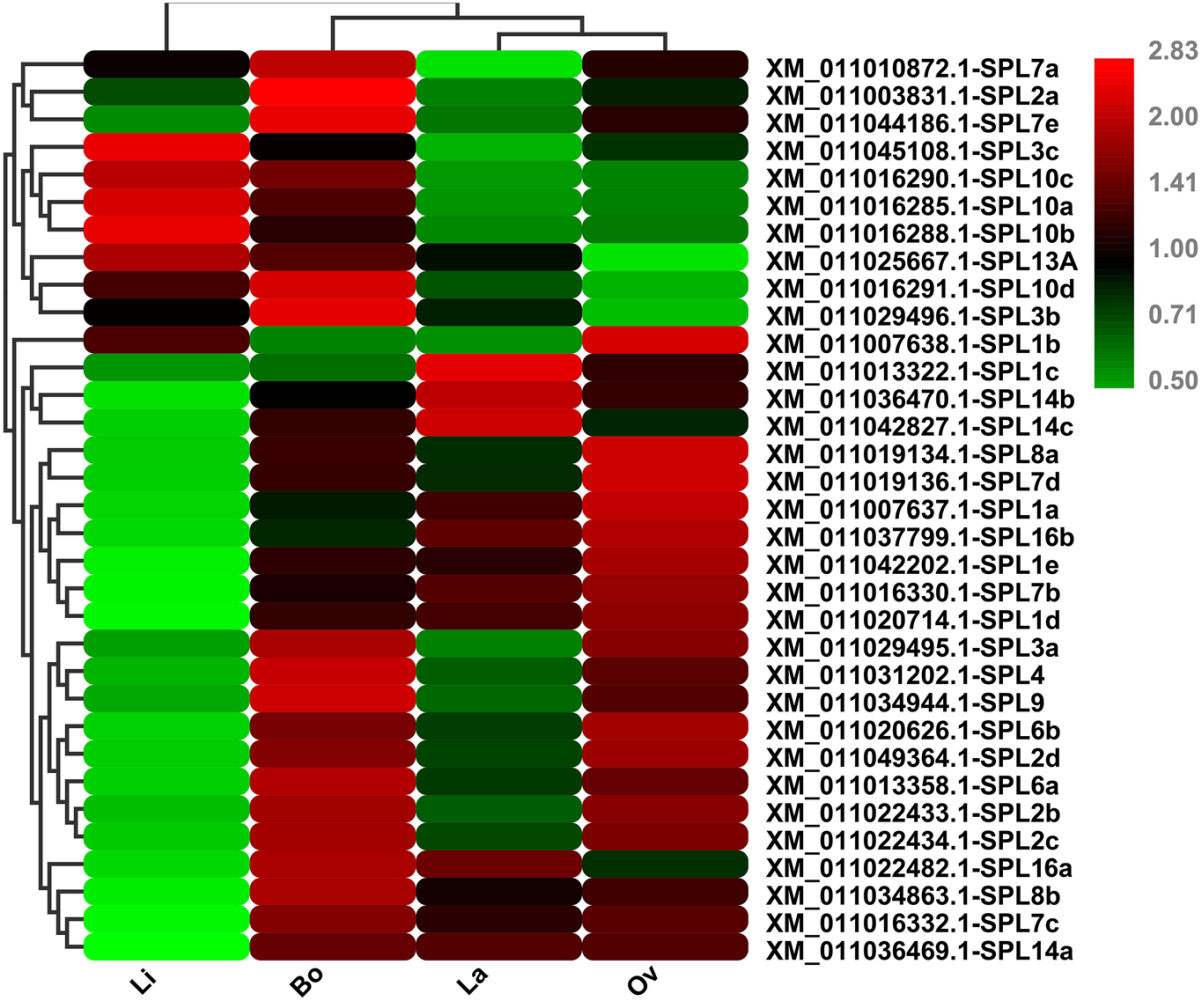
Heatmap representation of SPL gene expression in *P.* hl. FPKM values were identified from RNA-seq data and normalized by log_2_ transformation. The color scale represents log_2_-transformed values. Green represents low expression, and red represents high expression. This figure was generated with HEATMAP (https://www.omicshare.com/tools/Home/Soft/heatmap).

The RNA sequencing data was used to analyze the transcript levels of the putative SPL genes among the different *P. euphratica* leaves. A heat map was constructed to assess the expression profiles of the SPL genes based on the FPKM values (Fig. 3). All of the 33 SPL genes were widely expressed among the four *P. euphratica* heteromorphic leaves (Li, La, Ov, and Bo). The expression level was similar in La and Ov leaves. Twenty-four SPL TFs showed down-regulation in the Li, and 23 showed up-regulation in Bo leaves. Polarization of expression profiles in the two extreme leaf shapes (Li and Bo) indicated that SPL TF activity in Bo leaves was much higher than that in the Li leaves in the *P. euphratica* (Fig. 3).

### Functional identification and interaction network of the SPL TFs in *P*. hl

**Figure 4:**
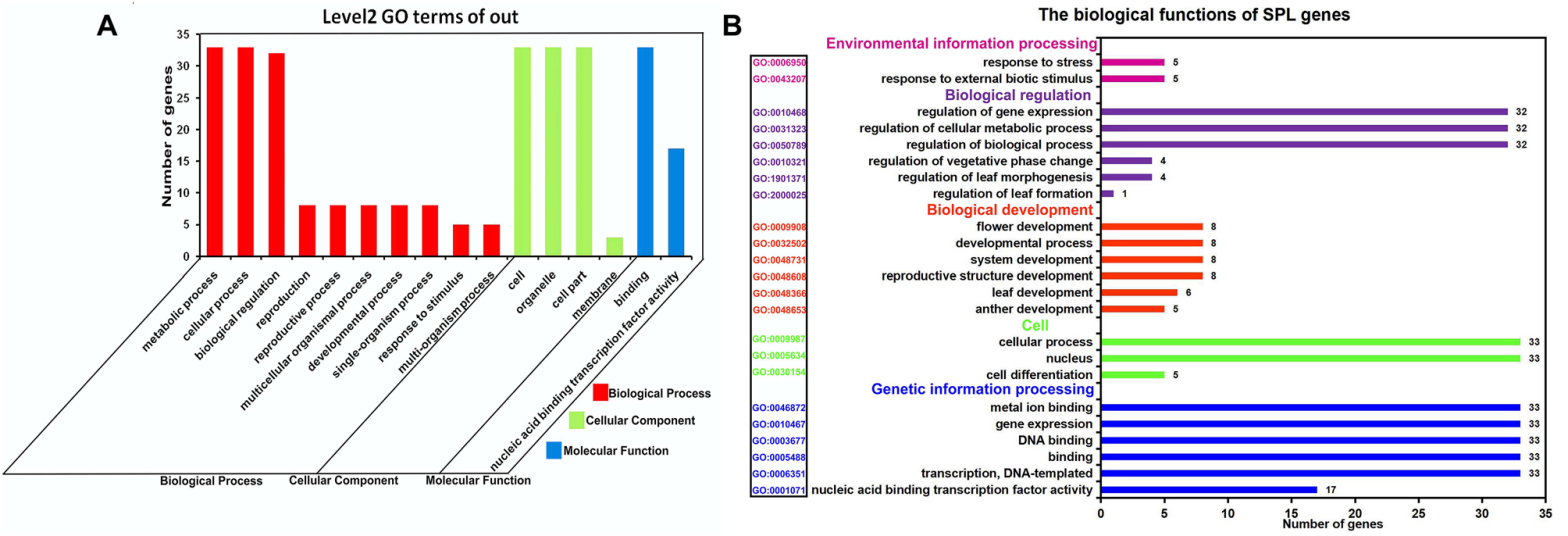
GO annotation of identified SPL TFs in three categories: biological processes, cellular components, and molecular functions. (**A**) GO enrichment. (**B**) Prediction of biological functions. This figure was generated by gogseasenior (https://www.omicshare.com/tools/Home/Soft/gogseasenior).

PeuSPLs function was re-annotated by GO annotation. The 33 PeuSPL genes were assigned to all three categories of biological functions, including biological process, cellular components, and molecular function (Fig. 4A). There were seventeen PeuSPL genes found with the ability to bind to specific DNA sequences in the nucleus that affected the transcription efficiency (GO: 0001071). Thirty-two PeuSPL genes might be involved in the regulation of biological processes (GO: 0050789), of which eight PeuSPL genes might be associated with developmental processes (GO: 0032502), such as regulation of vegetative phase change (GO: 0010321), flower development (GO: 0009908), and leaf development (GO: 0048366). Additionally, the 33 PeuSPL genes might be closely related to metal ions (GO: 0046872). Compared with the transcription factor database, the function of PeuSPL TFs was similar to the GO enrichment result (Fig. 4B).

**Figure 5:**
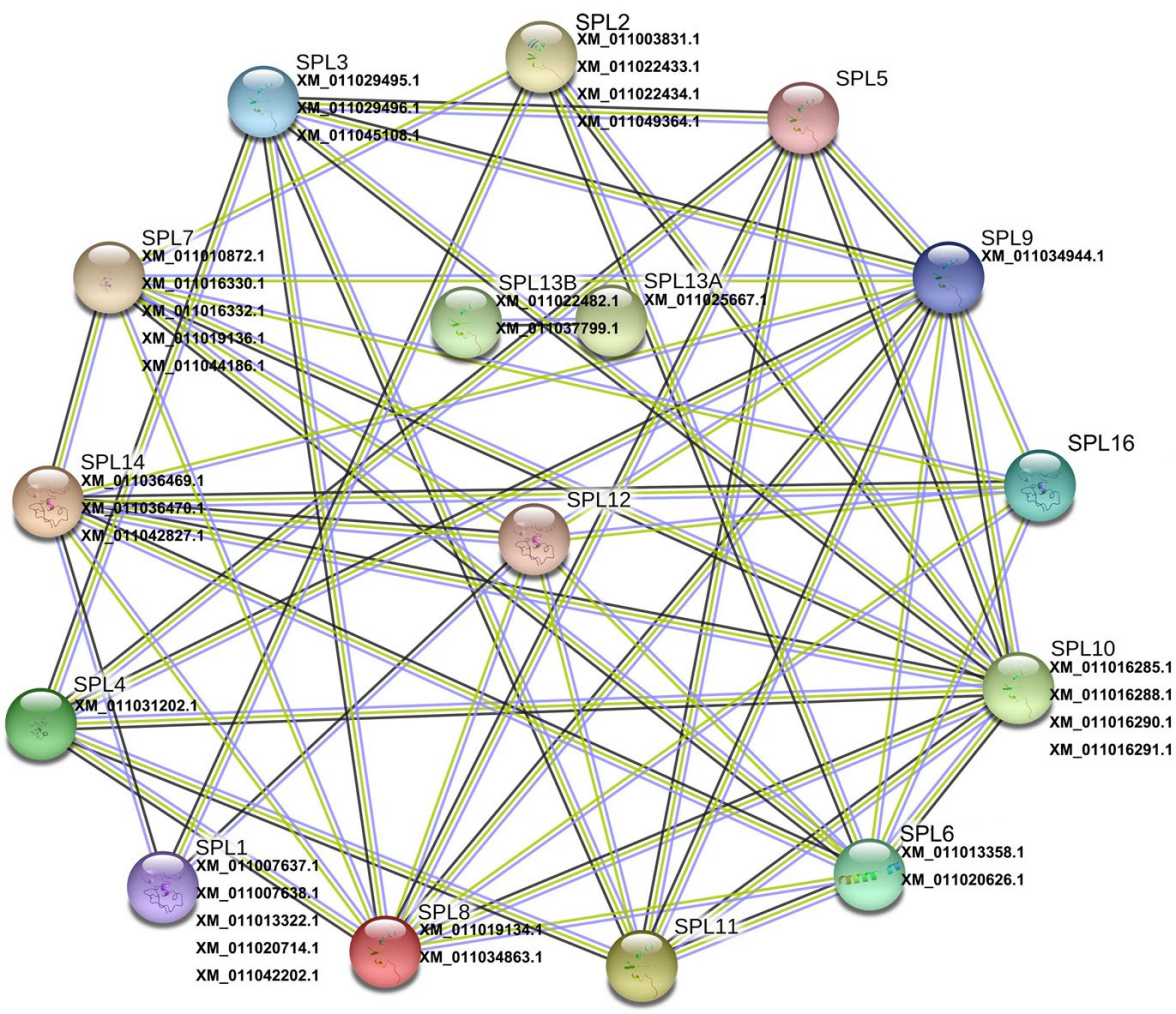
Functional interaction networks of SPL proteins in *P.* hl based on *Arabidopsis* orthologs*.* Nodes represent proteins, filled nodes shows the proteins with known or predicted 3D structure, empty nodes present the proteins whose 3D structure is unknown. Edges represent protein–protein association, black edges represent co-expression interactions, yellow edges represent textmining interaction, blue edges represent protein homology. This figure was generated by STRING (https://string-db.org/).

Further to understand the interaction between SPL proteins in the *P*. hl, an interaction network was constructed using STRING network analysis tool based on the *Arabidopsis* orthologs (Fig. 5). It can be seen from Fig. 5, the 3D structure is known or predicted of remain 15 SPL proteins, except SPL13A. The main interaction among these SPL proteins is co-expression, textmining and protein homology. Among them, SPL9 (*XM-011034944.1*) has maximum interaction, co-expression with SPL3, SPL4, SPL5, SPL8, SPL10 and SPL11; textmining and homology with SPL3, SPL4, SPL5, SPL6, SPL7, SPL8, SPL10, SPL11, SPL12, SPL14 and SPL16. SPL13A and SPL13B have minimum interaction, they only interact with each other (Fig. 5).

### Determination of regulatory relationships between RNAs in *P*. hl morphogenesis

**Figure 6:**
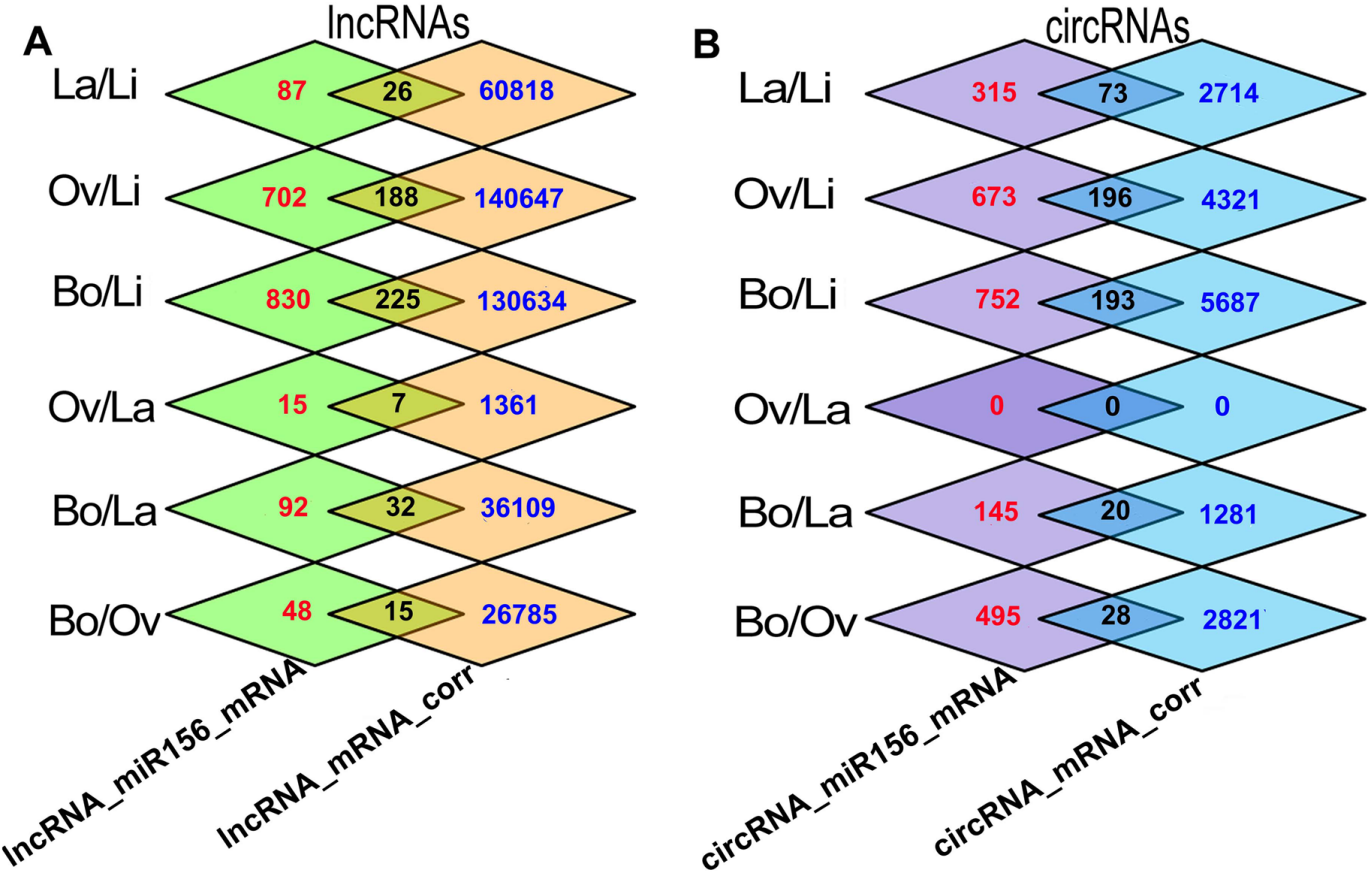
Regulatory relationships in *P*. hl*.* lncRNA–miR156–mRNA and circRNA–miR156–mRNA represent the number of lncRNA–mRNA and circRNA–mRNA relationships have same MREs of miR156 respectively. lncRNA–mRNA corr and circRNA–mRNA corr represent the number of lncRNA- mRNA and circRNA–mRNA relationships which showed positive correlations in expression trend. Interaction represents the number of met both two conditions. This figure was generated by Photoshop.

The regulatory relationships among non-coding RNAs (ncRNAs) and mRNAs in Li, La, Ov, and Bo leaves were predicted by MREs (The detail of interactions of miRNA156/SPL, miRNA156/lncRNAs and miRNA156/circRNAs are displayed in Table S4) and expression trend correlations are presented in Fig. 6A. For example, based on the same MRE of miR156, 113 differentially expressed lncRNA–mRNA relationships were constructed in La/Li, and according to the expression trend correlation, 60,844 lncRNA–mRNA relationships were identified. Based on their intersection, 26 lncRNA–miR156–mRNA regulatory relationships were constructed. Therefore, we identified 188, 225, 7, and 32 lncRNA–miRNA–mRNA regulatory relationships in Ov/Li, Bo/Li, Bo/La, and Bo/Ov comparisons, respectively (Fig. 6A). Moreover, the differentially expressed circRNA–miRNA–mRNA regulatory relationships were constructed using the same method, 73, 196, 193, 20 and 28 circRNA–miR156–mRNA regulatory relationships were determined in La/Li, Ov/Li, Bo/Li, Bo/La, and Bo/Ov, respectively (Fig. 6B).

### Regulatory networks of SPL TFs in *P*. hl

**Figure 7:**
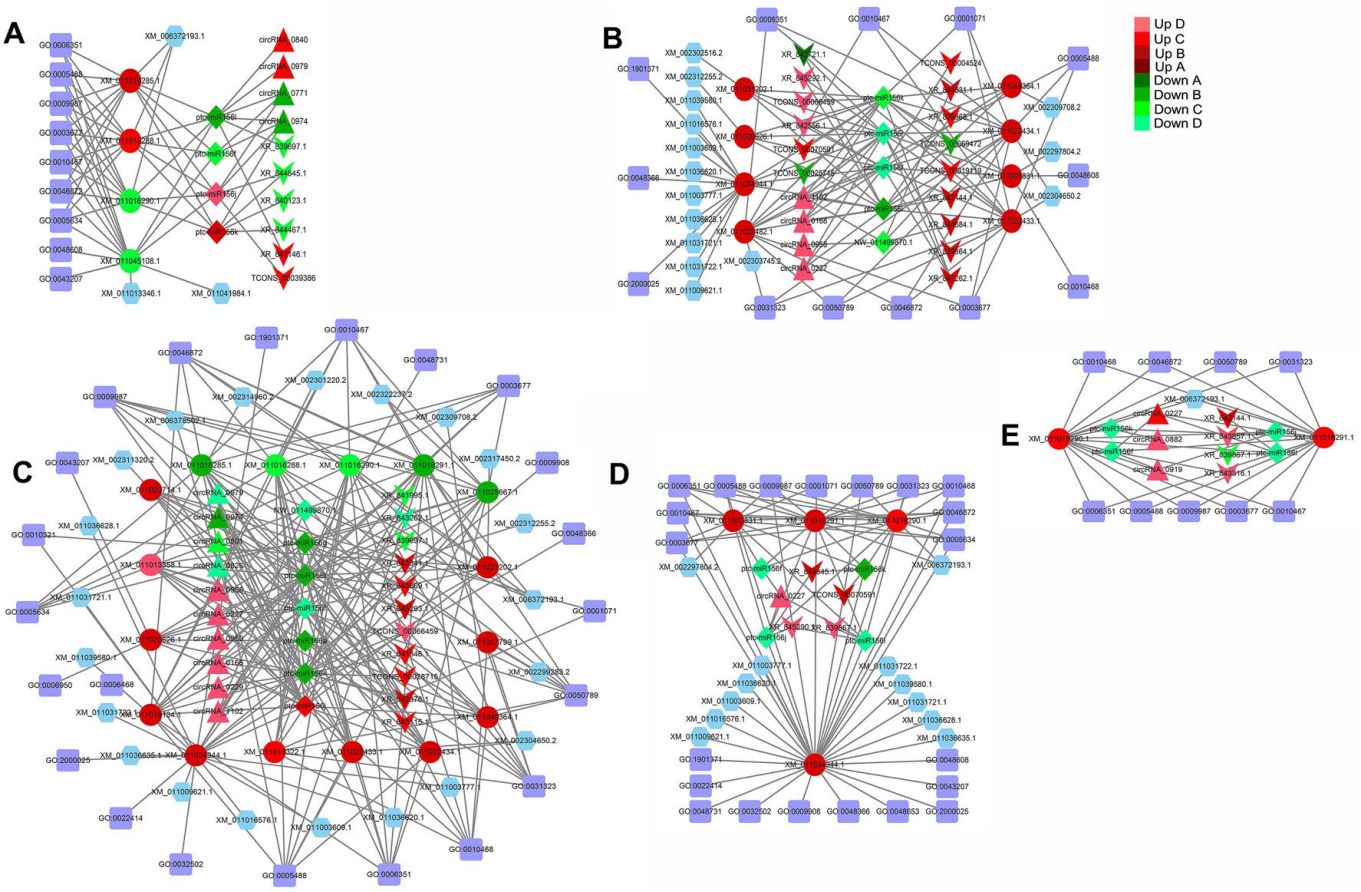
Regulatory networks and functional predictions of ncRNAs and mRNAs (SPL TFs) in the 5 sample pairs of *P. euphratica.* (**A**–**E**) show La/Li, Ov/Li, Bo/Li, Bo/La, and Bo/Ov sample pairs, respectively. The purple squares represent GO IDs; the diamonds, triangles, Vs, ellipses, and hexagons represent miRNAs, circRNAs, lncRNAs, SPL TFs, and target genes, respectively. As indicated in the upper right corner, red indicates up-regulated expression, among them twofold ≥ Up A > onefold, fivefold ≥ Up B > twofold, tenfold ≥ Up C > fivefold, Up D > tenfold; green indicates down-regulated expression, among them twofold ≥ Down A > onefold, fivefold ≥ Down B > twofold, tenfold ≥ Down C > fivefold, Down D > tenfold. This figure was generated by Cytoscape 3.8.2 (https://cytoscape.org/).

A regulatory network of the ceRNA (circRNA, lncRNA)–miRNA–SPL (mRNA) was established using Cytoscape. A total of 33 lncRNAs and 14 circRNAs interacted with miR156 in the five sample pairs (La/Li, Ov/Li, Bo/Li, Bo/La, and Bo/Ov) of *P.* hl, which regulated the SPL TF expression. For Bo/Ov, Ov/Li, Bo/Li, Bo/La and Bo/Ov, there were 4 circRNAs, 6 lncRNAs and 4 miRNAs co-regulated 4 SPL genes; 4 circRNAs, 15 lncRNAs and 5 miRNAs co-regulated 8 SPL genes; 10 circRNAs, 11 lncRNAs and 7 miRNAs co-regulated 17 SPL genes; 1 circRNA, 4 lncRNAs and 4 miRNAs co-regulated 4 SPL genes; and 3 circRNAs, 4 lncRNAs and 4 miRNAs co-regulated 2 SPL genes (Fig. 7). The complex regulatory networks showed the SPL TFs may play an important role in *P.* hl morphogenesis. It can be seen from Fig. 7 that PeuSPL9 (*XM-011034944.1*) and PeuSPL2 (*XM-011003831.1*, *XM-011022433.1*, *XM-011022434.1*, *XM-011049364.1*) were involved in leaf shape development (GO: 0048366) (Fig. 7B). PeuSPL13A (*XM-011025667.1*) can regulate metal ions (GO: 0046872). Moreover, PeuSPL4 (*XM-011031202.1*) and PeuSPL9 (*XM-011034944.1*) co-regulate photoperiod induction and are involved in the transition from juvenile to adult vegetative phase (GO: 0010321). PeuSPL8 (*XM-011019134.1*) can regulate signal transduction (GO: 0006468) (Fig. 7C).

In addition, circRNAs and lncRNAs were also involved in the miR156-SPL model in *P.* hl (Fig. 7). For example, the up-regulated expression of circRNA-0227 and four lncRNAs (*XR-839867.1*, *XR-844845.1*, *XR-845290.1*, and *TCONS-00070591*) down-regulates expression of four miRNAs (*ptc-miR156j*, *ptc-miR156k*, *ptc-miR156l*, and *ptc-miR156f*) in Bo/La, that leads to the up-regulation downstream TFs PeuSPL2 (*XM-011003831.1*), PeuSPL10 (*XM-011034944.1*, *XM-011016291.1*), PeuSPL9 (*XM-011034944.1*) and their downstream target genes (*XM-011003777.1*) in Bo/La (Fig. 7D).

### Gene expression validation in *P*. hl

**Figure 8:**
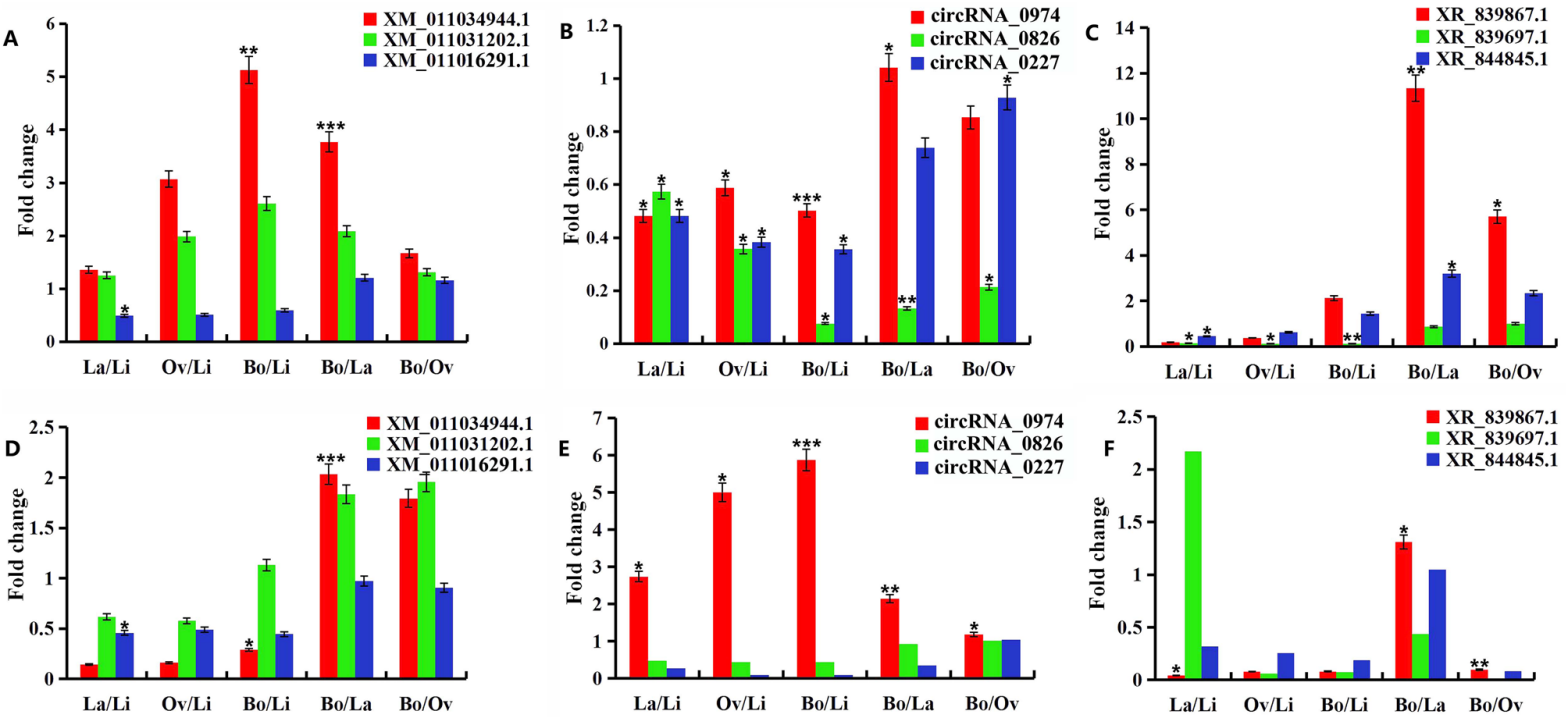
qPCR validation of the three kinds of RNAs in Li, La, Ov, and Bo leaves. (**A**–**C**) display the sequencing results; *P < 0.05, **P < 0.01, and ***P < 0.001 represent significant comparisons of sample pairs obtained using DESeq2. (**D**–**F**) display the corresponding RNA qPCR results; *P < 0.05, **P < 0.01, and ***P < 0.001 are the significance values of the comparison of sample pairs (For example Bo/Li) obtained by ‘f-test and t-test with EXCEL. Error bars indicate ± SD. This figure was generated by EXCEL.

The expression profiles of randomly selected RNAs, including SPL TFs (*XM-011034944.1*, *XM-011031202.1* and *XM-011016291.1*), circRNAs (*circRNA-0979*, *circRNA-1102*, and *circRNA-0168*), and lncRNAs (*XR-839867.1*, *XR-839697.1*, and *XR-844845.1*) were verified by qPCR. Furthermore, 18S RNA was used as reference genes for ceRNAs (circRNAs, lncRNAs) and mRNAs. The expression pattern of these RNAs was found to be similar to the sequencing results (Fig. 8). Therefore, the results of chain-specific sequencing results in this study were reliable.

## Discussion

The SPL family is plant-specific TFs that play various roles during plant growth and development^34^. In this study, we identified and characterized 33 genes that encode SPL proteins in *P. euphratica.* Sequence analysis of SPL genes in *P. euphratica* showed that they can be divided into two subgroups based on the presence of different motif(s) (Fig. 1). It was previously reported that gene duplication can also produce new functions^35^, these PeuSPL genes may have a variety of functions in *P. euphratica.* Motifs analyses indicated that the SPL proteins in the same subgroup had similar motifs, but had significant differences among different subgroups (Fig. 2A, B). Moreover, motifs 1 and motifs 2 existed nearly in each *P. euphratica* SPL protein, which indicated that they were conserved domains and played important roles in the *P. euphratica* SPL family. SPL proteins of these subgroups likely have special functions, due to the variation of motifs in different subgroups that indicated functional differentiation of the SPL TFs.

**Figure 9:**
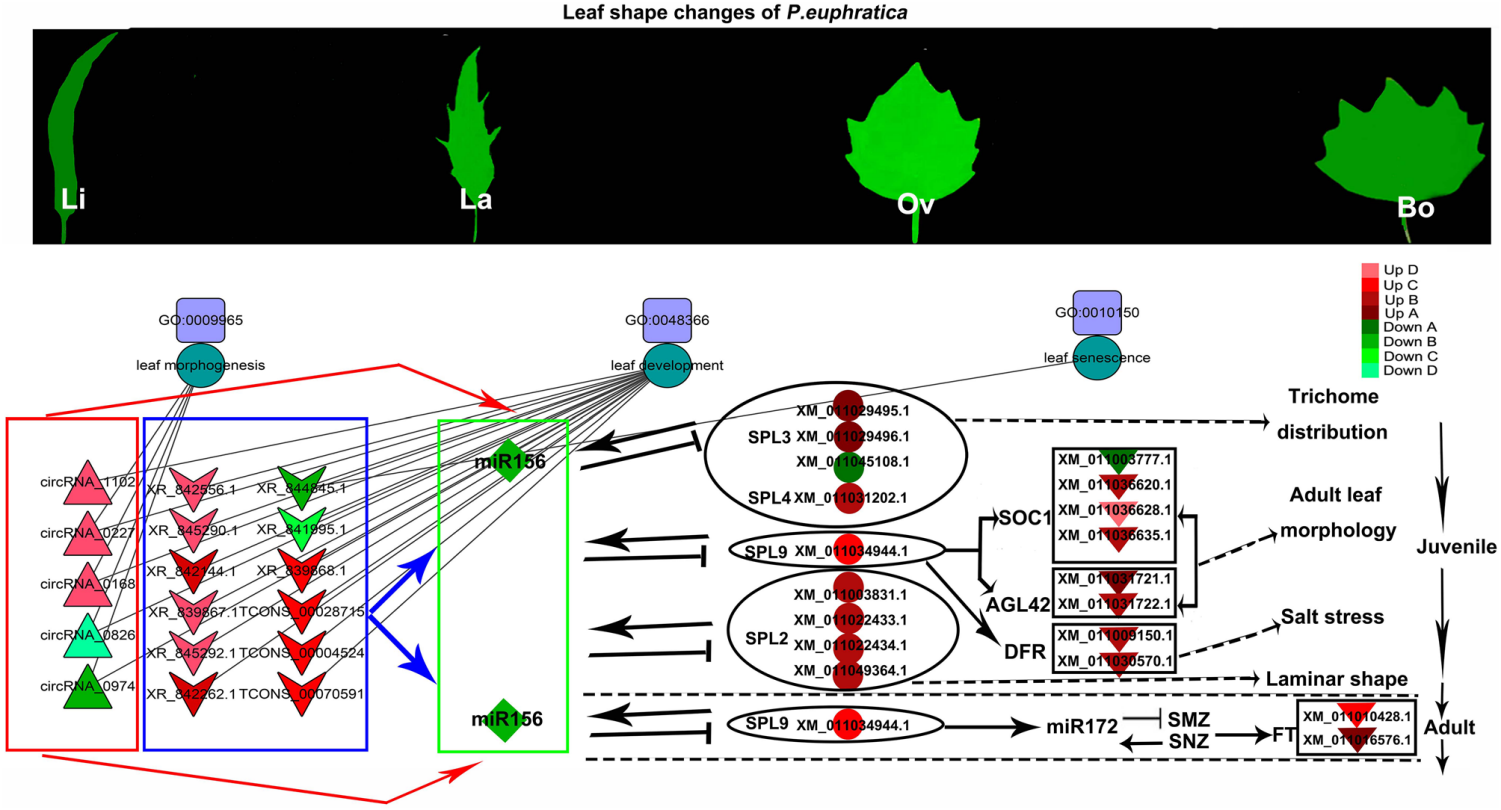
The interplay of SPL TFs and miRNAs in *P.* hl. Lines with arrowheads represent positive regulation, whereas lines with a bar at the end represent negative regulation. Triangles, Vs, rectangles, ellipses, and inverted triangles represent circRNAs, lncRNAs, GO IDs, biological processes, and target genes, respectively. As indicated in the right, red shows up-regulated expression, among them twofold ≥ Up A > onefold, fivefold ≥ Up B > twofold, tenfold ≥ Up C > fivefold, Up D > tenfold; green indicates down-regulated expression, among them twofold ≥ Down A > onefold, fivefold ≥ Down B > twofold, tenfold ≥ Down C > fivefold, Down D > tenfold. This figure was generated by Cytoscape 3.8.2 (https://cytoscape.org/) and Photoshop.

miR156-SPL module act as a regulatory hub in plant transition from juvenile to adult phases, and regulates leaf shape development and salt tolerance^14,36–38^. In this study, it was found that the expression level of miR156 declined from Li to Bo leaves (Fig. 7); and the expression levels of major SPL family members gradually increase from Li to Bo leaves (Figs. 3, 7). As mentioned earlier, the *P. euphratica* leaves of young tree are all Li, and later, it generates La, Ov, Bo leaves with increasing tree age. Therefore, this result indicated the same expression trend of miR156 and SPL with other plants and proved this model is evolutionarily conserved in plants. The functions of some PeuSPLs were same as in *Arabidopsis.* For example, overexpressed AtSPL10, AtSPL11, and AtSPL2 can promote serrated leaf genesis^39^; in this study, PeuSPL2 (*XM-011003831.1*, *XM-011022433.1*, *XM-011022434.1*, *XM-011049364.1*) expression was up-regulated in Bo leaves (Fig. 3). The margin of Li leaves is smooth, but is serrated in Bo leaves^40^. This result shows that PeuSPL2 should be responsible for serrated leaf genesis in *P. euphratica*, and may have similar functions as AtSPL2. However, for leaf index regulation and salt tolerance, the functions of some PeuSPL proteins may have differed from those of *Arabidopsis*. It has also been reported that miR156 overexpression enhances salt stress tolerance in *Arabidopsis* by the miR156–SPL9–DFR pathway, and young *Arabidopsis* have relatively strong resistance to adverse conditions^41^. However, for *P. euphratica*, miR156 was down-regulated in the Bo leaves (adult tree) and PeuSPL9 (*XM-011034944.1*) showed higher expression (Figs. 3, 7). A previous study found that adult tree of *P. euphratica* has stronger resistance to salt stress and adverse conditions^42^. These results indicated that the miR156–SPL9–DFR pathway works in different ways in these two plant species. In *Arabidopsis*, down-regulated miR156 or up-regulated SPL2, SPL9, SPL10, and SPL11 could cause leaves to become narrower^37,43^; however, in *P*. *x canadensis*, down-regulated miR156 causes leaves to become broad^44^. In this study also, down-regulated miR156 or up-regulated PeuSPL2 and PeuSPL9 caused leaves to become broad (Figs. 3, 9), this indicated that the function of PueSPL2 and PeuSPL9 was similar to that of *P*. *x canadensis,* but might was contrasting adverse with that of *Arabidopsis.* Moreover, AtSPL3, AtSPL4, and AtSPL5 overexpression also accelerated adult leaf abaxial trichome production^16^; however, there are no trichome in leaves of *P. euphratica*. Overall these results show that the miR156-SPL pathway plays key roles in *P.* hl salt tolerance and morphogenesis, but this pathway can directly regulate the transition from juvenile to adult phases only, and the vegetative phase change can affect salt tolerance and organ development. Hence it can be hypothesized that the miR156-SPL pathway might regulate leaf shape development and tolerance to stress indirectly.

According to ceRNA hypothesis, circRNAs, and lncRNAs can decoy miRNAs to affect the expression of target genes that have same MRE^19^. In this study, it was found that 14 circRNAs, and 33 lncRNAs may be involved in the miR156-SPL pathway in *P. euphratica* and affected leaf development (Figs. 7, 9). For example, *circRNA-0168* and *XR-845292.1* (lncRNA)might could promote PeuSPL9 (*XM-011034944.1*) expression by decoying miR156 (Fig. 7B and C); PeuSPL9 could activate the transcription of *XM-011036628.1* (*soc1*) and *XM-011031722.1* (AGL42) (Fig. 9). *Soc1* and *Soc1*-like genes play major role in the transition from vegetative to reproductive development^43^ and *soc1* is activated by an age-dependent mechanism^45^. *Soc1* and *agl42* were found to be high expressed in leaves and flowers^46,47^, thus in *P. euphratica* too, leaf shape was an age-dependent mechanism^2^. Therefore it was suspected that *soc1* and *agl42* are important in the morphogenesis of *P.* hl.Additionally, Li confirmed that circRNA-0168 expression was negatively correlated with miR156 families^1^, which indicated that the ceRNA (circRNA, lncRNA)–miR156–SPL9 (*XM-011034944.1*) plays an critical role in the miR156-SPL pathway.

## Conclusion

SPL TFs are widely distributed among higher plants and play critical roles in plant growth and development, and are also important in response to biotic and abiotic stresses. However, there was a lack of information on the SPL TFs in *P.* hl. To reveal the status of SPLs in *P*. hl, 33 SPL genes were identified and characterized in *P.* hl. Based on the phylogenetic relationships and comparisons with the well-studied SPLs of *Arabidopsis*, the function of PeuSPLs were predicted, and we found that miR156, 33 lncRNAs, and 14 circRNAs might be involved in expression regulation of the PeuSPL family, SPL TFs might play a key roles in *P*. hl morphogenesis, but they likely work in an indirect rather than direct manner.

## Supplementary Information

Supplementary Figure S1.

Supplementary Figure S2.

Supplementary Table S1.

Supplementary Table S2.

Supplementary Table S3.

Supplementary Table S4.

## Data Availability

All the raw data of RNA-Seq and small RNA sequencing have been submitted to GEO under accession numbers GSE120818 (RNA-Seq), GSE120821 (miRNA-Seq).
