# Supplementary material for: Identification and regulatory network analysis of SPL family transcription factors in Populus euphratica Oliv. heteromorphic leaves
Source: Sci Rep. 2022 Feb 21;12:2856. doi: 10.1038/s41598-022-06942-w (PMC8861001; doi:10.1038/s41598-022-06942-w)
Supplement: Supplementary file 1 — Supplementary Figure S1. [file 41598_2022_6942_MOESM1_ESM.pdf]

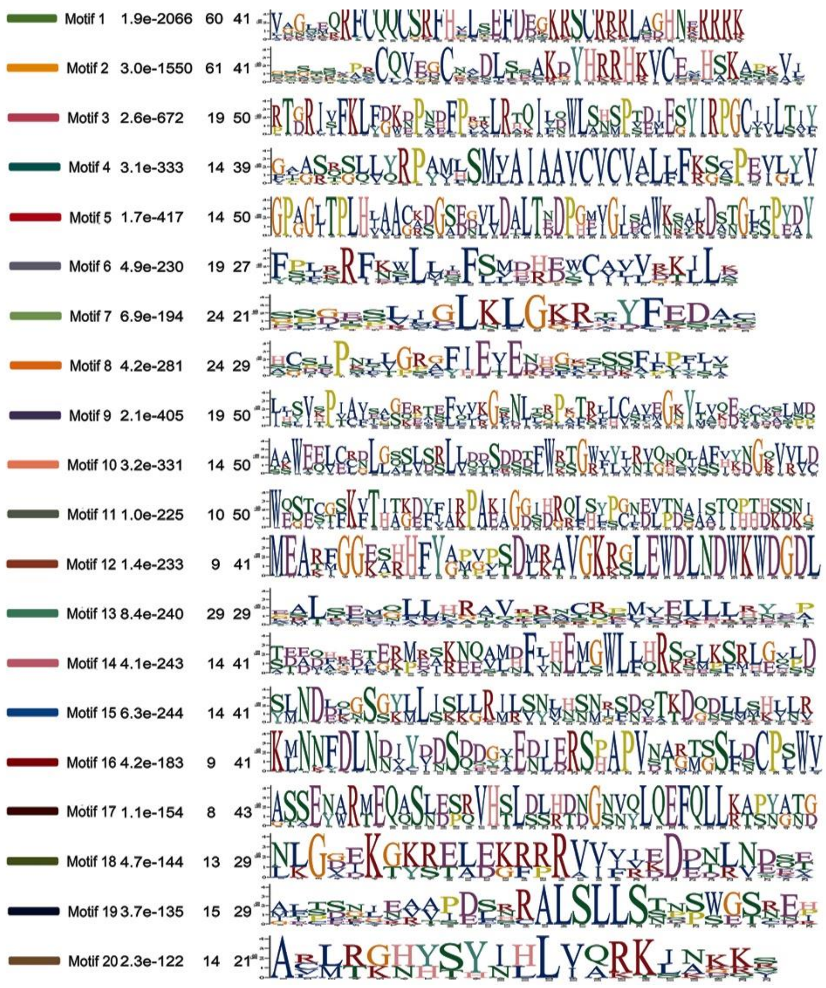

Figure S1. The conserved motifs were identified from poplar SPL proteins by MEME tool. Three columns of numbers indicated E-value, motif sites, the sequence width of motifs. This figure was generated by MEME 5.0.1(http://meme-suite.org/).
