# Supplementary material for: Identification and regulatory network analysis of SPL family transcription factors in Populus euphratica Oliv. heteromorphic leaves
Source: Sci Rep. 2022 Feb 21;12:2856. doi: 10.1038/s41598-022-06942-w (PMC8861001; doi:10.1038/s41598-022-06942-w)
Supplement: Supplementary file 3 — Supplementary Table S1. [file 41598_2022_6942_MOESM3_ESM.doc]

**Table S1. The primers of randomly selected RNAs were used in qPCR**

| **RNAs*** | **Primers** | **Sequence (5'-3')** | **product** | **Note** |
| --- | --- | --- | --- | --- |
| **18s** | **Forward** | **GTGGTGGTGCATGGCCGTTC** | **80** | **mRNA** |
|  | **Reverse** | **TAGCAGGCTGAGGTCTCGTTCG** |  |  |
| **circRNA_0974** | **Forward** | **CCTTGGGTCACCACCATTCA** | **111** | **circRNA** |
|  | **Reverse** | **AAGAGAGAGGGATCGGCAGT** |  |  |
| **circRNA_0826** | **Forward** | **GTGAAGGGCAAGTCACGTCT** | **122** | **circRNA** |
|  | **Reverse** | **GCCTTGACTGCCTCCAAAAC** |  |  |
| **circRNA_0227** | **Forward** | **ATCTCGCCGGTTGAATCCTC** | **148** | **circRNA** |
|  | **Reverse** | **CTGAAGCGTTGTCCTGGCTA** |  |  |
| **XM_011034944.1** | **Forward** | **AGAGCGGAACTGGGTCATCCTC** | **85** | **mRNA** |
|  | **Reverse** | **CTTGGTGGCTGCTGGTGCTG** |  |  |
| **XM_011031202.1** | **Forward** | **TGCGAGGTTCATGCGAAGTCAC** | **138** | **mRNA** |
|  | **Reverse** | **GTGTCCTGCCAAACGCCTACG** |  |  |
| **XM_011016291.1** | **Forward** | **GTTGCCAGGCAGGACGGATAAC** | **161** | **mRNA** |
|  | **Reverse** | **TGGATGGTGAAGACGGCAATGATG** |  |  |
| **XR_839697.1** | **Forward** | **TATATAGTCGCCCACCCGCC** | **126** | **lncRNA** |
|  | **Reverse** | **GCAGAGCGATCTAGCTGAACC** |  |  |
| **XR_839867.1** | **Forward** | **TGTGACAAGGATGGTGACAACAGG** | **94** | **lncRNA** |
|  | **Reverse** | **TTGGTCCGAGCAGTCAAGTGATG** |  |  |
| **XR_844845.1** | **Forward** | **CACGAAGTGGCAGTGGATGTAGC** | **92** | **lncRNA** |
|  | **Reverse** | **GCGATGGCATTCACGCTTGTAAG** |  |  |

**RNAs* include mRNA, miRNA and circRNA.**
