# Supplementary material for: Identification and regulatory network analysis of SPL family transcription factors in Populus euphratica Oliv. heteromorphic leaves
Source: Sci Rep. 2022 Feb 21;12:2856. doi: 10.1038/s41598-022-06942-w (PMC8861001; doi:10.1038/s41598-022-06942-w)
Supplement: Supplementary file 4 — Supplementary Table S2. [file 41598_2022_6942_MOESM4_ESM.doc]

| **mRNAs** | **Genes** | **Pfam** | **Domain** | **Interval** | **E-value** |
| --- | --- | --- | --- | --- | --- |
| **XM_011003831.1** | **SPL2a** | [**pfam03110**](https://www.ncbi.nlm.nih.gov/Structure/cdd/cddsrv.cgi?ascbin=8&maxaln=10&seltype=2&uid=pfam03110) | **SBP** | **1430-1654** | **5.65E-45** |
| **XM_011007637.1** | **SPL1a** | [**pfam03110**](https://www.ncbi.nlm.nih.gov/Structure/cdd/cddsrv.cgi?ascbin=8&maxaln=10&seltype=2&uid=pfam03110) | **SBP** | **1051-1275** | **9.57E-47** |
| **XM_011007638.1** | **SPL1b** | [**pfam03110**](https://www.ncbi.nlm.nih.gov/Structure/cdd/cddsrv.cgi?ascbin=8&maxaln=10&seltype=2&uid=pfam03110) | **SBP** | **1051-1275** | **7.32E-46** |
| **XM_011010872.1** | **SPL7a** | [**pfam03110**](https://www.ncbi.nlm.nih.gov/Structure/cdd/cddsrv.cgi?ascbin=8&maxaln=10&seltype=2&uid=pfam03110) | **SBP** | **478-702** | **6.44E-39** |
| **XM_011013322.1** | **SPL1c** | [**pfam03110**](https://www.ncbi.nlm.nih.gov/Structure/cdd/cddsrv.cgi?ascbin=8&maxaln=10&seltype=2&uid=pfam03110) | **SBP** | **1037-1261** | **3.22E-42** |
| **XM_011013358.1** | **SPL6a** | [**pfam03110**](https://www.ncbi.nlm.nih.gov/Structure/cdd/cddsrv.cgi?ascbin=8&maxaln=10&seltype=2&uid=pfam03110) | **SBP** | **642-866** | **3.63E-44** |
| **XM_011016285.1** | **SPL10a** | [**pfam03110**](https://www.ncbi.nlm.nih.gov/Structure/cdd/cddsrv.cgi?ascbin=8&maxaln=10&seltype=2&uid=pfam03110) | **SBP** | **1244-1468** | **1.12E-44** |
| **XM_011016288.1** | **SPL10** | [**pfam03110**](https://www.ncbi.nlm.nih.gov/Structure/cdd/cddsrv.cgi?ascbin=8&maxaln=10&seltype=2&uid=pfam03110) | **SBP** | **1244-1468** | **5.53E-45** |
| **XM_011016290.1** | **SPL10b** | [**pfam03110**](https://www.ncbi.nlm.nih.gov/Structure/cdd/cddsrv.cgi?ascbin=8&maxaln=10&seltype=2&uid=pfam03110) | **SBP** | **1244-1468** | **6.12E-45** |
| **XM_011016291.1** | **SPL10d** | [**pfam03110**](https://www.ncbi.nlm.nih.gov/Structure/cdd/cddsrv.cgi?ascbin=8&maxaln=10&seltype=2&uid=pfam03110) | **SBP** | **1244-1468** | **4.6E-45** |
| **XM_011016330.1** | **SPL7b** | [**pfam03110**](https://www.ncbi.nlm.nih.gov/Structure/cdd/cddsrv.cgi?ascbin=8&maxaln=10&seltype=2&uid=pfam03110) | **SBP** | **480-704** | **1.53E-38** |
| **XM_011016332.1** | **SPL7c** | [**pfam03110**](https://www.ncbi.nlm.nih.gov/Structure/cdd/cddsrv.cgi?ascbin=8&maxaln=10&seltype=2&uid=pfam03110) | **SBP** | **478-702** | **1.52E-38** |
| **XM_011019134.1** | **SPL8a** | [**pfam03110**](https://www.ncbi.nlm.nih.gov/Structure/cdd/cddsrv.cgi?ascbin=8&maxaln=10&seltype=2&uid=pfam03110) | **SBP** | **803-1027** | **2.63E-43** |
| **XM_011019136.1** | **SPL7d** | [**pfam03110**](https://www.ncbi.nlm.nih.gov/Structure/cdd/cddsrv.cgi?ascbin=8&maxaln=10&seltype=2&uid=pfam03110) | **SBP** | **823-1044** | **7.12E-44** |
| **XM_011020626.1** | **SPL6b** | [**pfam03110**](https://www.ncbi.nlm.nih.gov/Structure/cdd/cddsrv.cgi?ascbin=8&maxaln=10&seltype=2&uid=pfam03110) | **SBP** | **685-909** | **2.31E-46** |
| **XM_011020714.1** | **SPL1d** | [**pfam03110**](https://www.ncbi.nlm.nih.gov/Structure/cdd/cddsrv.cgi?ascbin=8&maxaln=10&seltype=2&uid=pfam03110) | **SBP** | **984-1208** | **1.9E-41** |
| **XM_011022433.1** | **SPL2b** | [**pfam03110**](https://www.ncbi.nlm.nih.gov/Structure/cdd/cddsrv.cgi?ascbin=8&maxaln=10&seltype=2&uid=pfam03110) | **SBP** | **1360-1584** | **2.37E-44** |
| **XM_011022434.1** | **SPL2c** | [**pfam03110**](https://www.ncbi.nlm.nih.gov/Structure/cdd/cddsrv.cgi?ascbin=8&maxaln=10&seltype=2&uid=pfam03110) | **SBP** | **1356-1580** | **2.48E-44** |
| **XM_011022482.1** | **SPL16a** | [**pfam03110**](https://www.ncbi.nlm.nih.gov/Structure/cdd/cddsrv.cgi?ascbin=8&maxaln=10&seltype=2&uid=pfam03110) | **SBP** | **782-1006** | **1.05E-43** |
| **XM_011025667.1** | **SPL13A** | [**pfam03110**](https://www.ncbi.nlm.nih.gov/Structure/cdd/cddsrv.cgi?ascbin=8&maxaln=10&seltype=2&uid=pfam03110) | **SBP** | **1019-1243** | **6.84E-43** |
| **XM_011029495.1** | **SPL3a** | [**pfam03110**](https://www.ncbi.nlm.nih.gov/Structure/cdd/cddsrv.cgi?ascbin=8&maxaln=10&seltype=2&uid=pfam03110) | **SBP** | **322-546** | **9.65E-41** |
| **XM_011029496.1** | **SPL3b** | [**pfam03110**](https://www.ncbi.nlm.nih.gov/Structure/cdd/cddsrv.cgi?ascbin=8&maxaln=10&seltype=2&uid=pfam03110) | **SBP** | **332-526** | **2.73E-30** |
| **XM_011031202.1** | **SPL4** | [**pfam03110**](https://www.ncbi.nlm.nih.gov/Structure/cdd/cddsrv.cgi?ascbin=8&maxaln=10&seltype=2&uid=pfam03110) | **SBP** | **504-728** | **9.74E-43** |
| **XM_011034863.1** | **SPL8b** | [**pfam03110**](https://www.ncbi.nlm.nih.gov/Structure/cdd/cddsrv.cgi?ascbin=8&maxaln=10&seltype=2&uid=pfam03110) | **SBP** | **975-1199** | **3.68E-43** |
| **XM_011034944.1** | **SPL9** | [**pfam03110**](https://www.ncbi.nlm.nih.gov/Structure/cdd/cddsrv.cgi?ascbin=8&maxaln=10&seltype=2&uid=pfam03110) | **SBP** | **539-763** | **3.76E-45** |
| **XM_011036469.1** | **SPL14a** | [**pfam03110**](https://www.ncbi.nlm.nih.gov/Structure/cdd/cddsrv.cgi?ascbin=8&maxaln=10&seltype=2&uid=pfam03110) | **SBP** | **870-1094** | **8.16E-45** |
| **XM_011036470.1** | **SPL14b** | [**pfam03110**](https://www.ncbi.nlm.nih.gov/Structure/cdd/cddsrv.cgi?ascbin=8&maxaln=10&seltype=2&uid=pfam03110) | **SBP** | **869-1093** | **6.64E-44** |
| **XM_011037799.1** | **SPL16b** | [**pfam03110**](https://www.ncbi.nlm.nih.gov/Structure/cdd/cddsrv.cgi?ascbin=8&maxaln=10&seltype=2&uid=pfam03110) | **SBP** | **871-1095** | **3.2E-45** |
| **XM_011042202.1** | **SPL1e** | [**pfam03110**](https://www.ncbi.nlm.nih.gov/Structure/cdd/cddsrv.cgi?ascbin=8&maxaln=10&seltype=2&uid=pfam03110) | **SBP** | **1123-1347** | **3.38E-45** |
| **XM_011042827.1** | **SPL14c** | [**pfam03110**](https://www.ncbi.nlm.nih.gov/Structure/cdd/cddsrv.cgi?ascbin=8&maxaln=10&seltype=2&uid=pfam03110) | **SBP** | **832-1056** | **2.17E-43** |
| **XM_011044186.1** | **SPL7e** | [**pfam03110**](https://www.ncbi.nlm.nih.gov/Structure/cdd/cddsrv.cgi?ascbin=8&maxaln=10&seltype=2&uid=pfam03110) | **SBP** | **526-750** | **1.87E-46** |
| **XM_011045108.1** | **SPL3c** | [**pfam03110**](https://www.ncbi.nlm.nih.gov/Structure/cdd/cddsrv.cgi?ascbin=8&maxaln=10&seltype=2&uid=pfam03110) | **SBP** | **328-552** | **1.74E-38** |
| **XM_011049364.1** | **SPL2d** | [**pfam03110**](https://www.ncbi.nlm.nih.gov/Structure/cdd/cddsrv.cgi?ascbin=8&maxaln=10&seltype=2&uid=pfam03110) | **SBP** | **493-717** | **8.93E-42** |

**Table S2 Conserved domain distribution of SPL transcription factors in the *Populus euphratica* Oliv. heteromorphic leaves**
