# Supplementary material for: Identification and regulatory network analysis of SPL family transcription factors in Populus euphratica Oliv. heteromorphic leaves
Source: Sci Rep. 2022 Feb 21;12:2856. doi: 10.1038/s41598-022-06942-w (PMC8861001; doi:10.1038/s41598-022-06942-w)
Supplement: Supplementary file 5 — Supplementary Table S3. [file 41598_2022_6942_MOESM5_ESM.doc]

| **mRNAs** | **Gene** | **Number of amino acids** | **Molecular weight** | **Theoretical pI** | **Negative (Asp + Glu)** | **Positive (Arg + Lys)** | **Grand average of hydropathicity (GRAVY)** | **Instability index** |
| --- | --- | --- | --- | --- | --- | --- | --- | --- |
| **XM_011003831.1** | **SPL2a** | **444** | **48909.45** | **8.67** | **47** | **53** | **-0.699** | **53.66** |
| **XM_011007637.1** | **SPL1a** | **1002** | **110956.19** | **6.02** | **121** | **105** | **-0.354** | **53.08** |
| **XM_011007638.1** | **SPL1b** | **974** | **108123.92** | **6.02** | **120** | **104** | **-0.364** | **52.96** |
| **XM_011010872.1** | **SPL7a** | **791** | **88344.27** | **6.57** | **96** | **91** | **-0.27** | **47.07** |
| **XM_011013322.1** | **SPL1c** | **1034** | **115383.23** | **8.01** | **121** | **125** | **-0.408** | **47.08** |
| **XM_011013358.1** | **SPL6a** | **498** | **54667.54** | **8.27** | **53** | **57** | **-0.416** | **50.05** |
| **XM_011016285.1** | **SPL10a** | **472** | **51963.81** | **8.68** | **49** | **56** | **-0.61** | **62.24** |
| **XM_011016288.1** | **SPL10** | **465** | **51129.85** | **8.61** | **49** | **55** | **-0.613** | **63.89** |
| **XM_011016290.1** | **SPL10b** | **455** | **50024.55** | **8.58** | **48** | **54** | **-0.629** | **62.31** |
| **XM_011016291.1** | **SPL10d** | **448** | **49190.59** | **8.49** | **48** | **53** | **-0.632** | **63.49** |
| **XM_011016330.1** | **SPL7b** | **803** | **90140.96** | **6.21** | **101** | **90** | **-0.339** | **48.43** |
| **XM_011016332.1** | **SPL7c** | **794** | **88978.63** | **6.25** | **100** | **90** | **-0.34** | **48.4** |
| **XM_011019134.1** | **SPL8a** | **326** | **36309.95** | **9.11** | **28** | **35** | **-0.768** | **59.92** |
| **XM_011019136.1** | **SPL7d** | **401** | **44197.04** | **8.26** | **41** | **44** | **-0.606** | **55.18** |
| **XM_011020626.1** | **SPL6b** | **510** | **55765.47** | **6.99** | **53** | **52** | **-0.454** | **47.62** |
| **XM_011020714.1** | **SPL1d** | **1035** | **115441.2** | **7.99** | **122** | **126** | **-0.38** | **42.63** |
| **XM_011022433.1** | **SPL2b** | **485** | **53740.5** | **8.81** | **51** | **58** | **-0.758** | **51.52** |
| **XM_011022434.1** | **SPL2c** | **483** | **53570.3** | **8.81** | **51** | **58** | **-0.769** | **52.54** |
| **XM_011022482.1** | **SPL16a** | **313** | **34254.95** | **9.12** | **28** | **35** | **-0.712** | **52.5** |
| **XM_011025667.1** | **SPL13A** | **381** | **41870.53** | **9.09** | **30** | **39** | **-0.663** | **57.82** |
| **XM_011029495.1** | **SPL3a** | **148** | **16487.85** | **6.52** | **27** | **26** | **-1.352** | **67.95** |
| **XM_011029496.1** | **SPL3b** | **138** | **15225.55** | **8.21** | **23** | **25** | **-1.355** | **68.46** |
| **XM_011031202.1** | **SPL4** | **241** | **27057.43** | **9.03** | **33** | **41** | **-0.876** | **72.23** |
| **XM_011034863.1** | **SPL8b** | **328** | **36371.03** | **8.85** | **28** | **33** | **-0.722** | **59.31** |
| **XM_011034944.1** | **SPL9** | **382** | **40916.31** | **9.13** | **28** | **37** | **-0.61** | **59.52** |
| **XM_011036469.1** | **SPL14a** | **1073** | **118858.13** | **8.49** | **116** | **125** | **-0.507** | **58.78** |
| **XM_011036470.1** | **SPL14b** | **991** | **110255.43** | **8.51** | **110** | **119** | **-0.517** | **61.1** |
| **XM_011037799.1** | **SPL16b** | **376** | **41248.83** | **9.14** | **32** | **41** | **-0.653** | **54.43** |
| **XM_011042202.1** | **SPL1e** | **1004** | **111336.4** | **6.15** | **123** | **109** | **-0.416** | **53.23** |
| **XM_011042827.1** | **SPL14c** | **1072** | **118988.54** | **8.39** | **119** | **127** | **-0.45** | **57.65** |
| **XM_011044186.1** | **SPL7e** | **409** | **45360.17** | **8.63** | **37** | **42** | **-0.515** | **65.41** |
| **XM_011045108.1** | **SPL3c** | **144** | **16219.77** | **6.22** | **28** | **26** | **-1.184** | **86.65** |
| **XM_011049364.1** | **SPL2d** | **257** | **29252.4** | **9.49** | **27** | **40** | **-0.462** | **45.84** |

**Table S3 protein properties of SPL transcription factors in the *Populus euphratica* Oliv. heteromorphic leaves**
