# Supplementary material for: Identification and regulatory network analysis of SPL family transcription factors in Populus euphratica Oliv. heteromorphic leaves
Source: Sci Rep. 2022 Feb 21;12:2856. doi: 10.1038/s41598-022-06942-w (PMC8861001; doi:10.1038/s41598-022-06942-w)
Supplement: Supplementary file 6 — Supplementary Table S4. [file 41598_2022_6942_MOESM6_ESM.docx]

Table S4 miRNA156/SPL, miRNA156/lncRNAs and miRNA156/circRNAs interactions analysis by psRNATarget

| miRNA | Target | Expect-  ation | miRNA  start | miRNA  end | Target  start | Target  end | miRNA_aligned_fragment | Target_aligned_fragment | Inhibition model |
| --- | --- | --- | --- | --- | --- | --- | --- | --- | --- |
| ptc-miR156a/b/c/d/e/f | SPL10/XM_011016285.1 | 1 | 1 | 20 | 1857 | 1876 | UGACAGAAGAGAGUGAGCAC | GUGCUCUCUCUCUUCUGUCA | Cleavage |
| ptc-miR156a/b/c/d/e/f | SPL10/XM_011016288.1 | 1 | 1 | 20 | 1836 | 1855 | UGACAGAAGAGAGUGAGCAC | GUGCUCUCUCUCUUCUGUCA | Cleavage |
| ptc-miR156a/b/c/d/e/f | SPL10/XM_011016290.1 | 1 | 1 | 20 | 1806 | 1825 | UGACAGAAGAGAGUGAGCAC | GUGCUCUCUCUCUUCUGUCA | Cleavage |
| ptc-miR156a/b/c/d/e/f | SPL10/XM_011016291.1 | 1 | 1 | 20 | 1785 | 1804 | UGACAGAAGAGAGUGAGCAC | GUGCUCUCUCUCUUCUGUCA | Cleavage |
| ptc-miR156a/b/c/d/e/f | SPL13A/XM_011025667.1 | 1 | 1 | 20 | 1638 | 1657 | UGACAGAAGAGAGUGAGCAC | GUGCUCUCUCUCUUCUGUCA | Cleavage |
| ptc-miR156a/b/c/d/e/f | SPL13B/XM_011022482.1 | 1 | 1 | 20 | 1380 | 1399 | UGACAGAAGAGAGUGAGCAC | GUGCUCUCUCUCUUCUGUCA | Cleavage |
| ptc-miR156a/b/c/d/e/f | SPL13B/XM_011037799.1 | 1 | 1 | 20 | 1469 | 1488 | UGACAGAAGAGAGUGAGCAC | GUGCUCUCUCUCUUCUGUCA | Cleavage |
| ptc-miR156a/b/c/d/e/f | SPL2/XM_011003831.1 | 1 | 1 | 20 | 2028 | 2047 | UGACAGAAGAGAGUGAGCAC | GUGCUCUCUCUCUUCUGUCA | Cleavage |
| ptc-miR156a/b/c/d/e/f | SPL2/XM_011022433.1 | 1 | 1 | 20 | 1970 | 1989 | UGACAGAAGAGAGUGAGCAC | GUGCUCUCUCUCUUCUGUCA | Cleavage |
| ptc-miR156a/b/c/d/e/f | SPL2/XM_011022434.1 | 1 | 1 | 20 | 1960 | 1979 | UGACAGAAGAGAGUGAGCAC | GUGCUCUCUCUCUUCUGUCA | Cleavage |
| ptc-miR156a/b/c/d/e/f | SPL2/XM_011049364.1 | 2 | 1 | 20 | 879 | 898 | UGACAGAAGAGAGUGAGCAC | UUGCUCUCUCUCUUCUGUCA | Cleavage |
| ptc-miR156a/b/c/d/e/f | SPL3/XM_011029495.1 | 2 | 1 | 20 | 721 | 740 | UGACAGAAGAGAGUGAGCAC | AUGCUCUCUCUCUUCUGUCA | Cleavage |
| ptc-miR156a/b/c/d/e/f | SPL3/XM_011029496.1 | 2 | 1 | 20 | 701 | 720 | UGACAGAAGAGAGUGAGCAC | AUGCUCUCUCUCUUCUGUCA | Cleavage |
| ptc-miR156a/b/c/d/e/f | SPL3/XM_011045108.1 | 1 | 1 | 20 | 726 | 745 | UGACAGAAGAGAGUGAGCAC | AUGCUCUCUCUCUUCUGUCA | Cleavage |
| ptc-miR156a/b/c/d/e/f | SPL4/XM_011031202.1 | 2 | 1 | 20 | 894 | 913 | UGACAGAAGAGAGUGAGCAC | AUGCUCCCUCUCUUCUGUCA | Cleavage |
| ptc-miR156a/b/c/d/e/f | SPL6/XM_011013358.1 | 1 | 1 | 20 | 1195 | 1214 | UGACAGAAGAGAGUGAGCAC | GUGCUCUCUCUCUUCUGUCA | Cleavage |
| ptc-miR156a/b/c/d/e/f | SPL6/XM_011020626.1 | 1 | 1 | 20 | 1280 | 1299 | UGACAGAAGAGAGUGAGCAC | GUGCUCUCUCUCUUCUGUCA | Cleavage |
| ptc-miR156a/b/c/d/e/f | SPL7/XM_011019136.1 | 1 | 1 | 20 | 1130 | 1149 | UGACAGAAGAGAGUGAGCAC | GUGCUCUCUCUCUUCUGUCA | Cleavage |
| ptc-miR156a/b/c/d/e/f | SPL7/XM_011044186.1 | 1 | 1 | 20 | 833 | 852 | UGACAGAAGAGAGUGAGCAC | GUGCUCUCUCUCUUCUGUCA | Cleavage |
| ptc-miR156a/b/c/d/e/f | SPL9/XM_011034944.1 | 1 | 1 | 20 | 1095 | 1114 | UGACAGAAGAGAGUGAGCAC | GUGCUCUCUCUCUUCUGUCA | Cleavage |
| ptc-miR156g/h/i/j | SPL10/XM_011016285.1 | 1 | 1 | 21 | 1857 | 1877 | UUGACAGAAGAUAGAGAGCAC | GUGCUCUCUCUCUUCUGUCAA | Cleavage |
| ptc-miR156g/h/i/j | SPL10/XM_011016288.1 | 1 | 1 | 21 | 1836 | 1856 | UUGACAGAAGAUAGAGAGCAC | GUGCUCUCUCUCUUCUGUCAA | Cleavage |
| ptc-miR156g/h/i/j | SPL10/XM_011016290.1 | 1 | 1 | 21 | 1806 | 1826 | UUGACAGAAGAUAGAGAGCAC | GUGCUCUCUCUCUUCUGUCAA | Cleavage |
| ptc-miR156g/h/i/j | SPL10/XM_011016291.1 | 1 | 1 | 21 | 1785 | 1805 | UUGACAGAAGAUAGAGAGCAC | GUGCUCUCUCUCUUCUGUCAA | Cleavage |
| ptc-miR156g/h/i/j | SPL13A/XM_011025667.1 | 2 | 1 | 21 | 1638 | 1658 | UUGACAGAAGAUAGAGAGCAC | GUGCUCUCUCUCUUCUGUCAU | Cleavage |
| ptc-miR156g/h/i/j | SPL13B/XM_011022482.1 | 2 | 1 | 21 | 1380 | 1400 | UUGACAGAAGAUAGAGAGCAC | GUGCUCUCUCUCUUCUGUCAU | Cleavage |
| ptc-miR156g/h/i/j | SPL13B/XM_011037799.1 | 2 | 1 | 21 | 1469 | 1489 | UUGACAGAAGAUAGAGAGCAC | GUGCUCUCUCUCUUCUGUCAU | Cleavage |
| ptc-miR156g/h/i/j | SPL2/XM_011003831.1 | 1 | 1 | 21 | 2028 | 2048 | UUGACAGAAGAUAGAGAGCAC | GUGCUCUCUCUCUUCUGUCAA | Cleavage |
| ptc-miR156g/h/i/j | SPL2/XM_011022433.1 | 1 | 1 | 21 | 1970 | 1990 | UUGACAGAAGAUAGAGAGCAC | GUGCUCUCUCUCUUCUGUCAA | Cleavage |
| ptc-miR156g/h/i/j | SPL2/XM_011022434.1 | 1 | 1 | 21 | 1960 | 1980 | UUGACAGAAGAUAGAGAGCAC | GUGCUCUCUCUCUUCUGUCAA | Cleavage |
| ptc-miR156g/h/i/j | SPL2/XM_011049364.1 | 2 | 1 | 20 | 880 | 899 | UUGACAGAAGAUAGAGAGCA | UGCUCUCUCUCUUCUGUCAU | Cleavage |
| ptc-miR156g/h/i/j | SPL3/XM_011029495.1 | 1 | 1 | 20 | 722 | 741 | UUGACAGAAGAUAGAGAGCA | UGCUCUCUCUCUUCUGUCAA | Cleavage |
| ptc-miR156g/h/i/j | SPL3/XM_011029496.1 | 1 | 1 | 20 | 702 | 721 | UUGACAGAAGAUAGAGAGCA | UGCUCUCUCUCUUCUGUCAA | Cleavage |
| ptc-miR156g/h/i/j | SPL3/XM_011045108.1 | 1 | 1 | 20 | 727 | 746 | UUGACAGAAGAUAGAGAGCA | UGCUCUCUCUCUUCUGUCAA | Cleavage |
| ptc-miR156g/h/i/j | SPL4/XM_011031202.1 | 2 | 1 | 20 | 895 | 914 | UUGACAGAAGAUAGAGAGCA | UGCUCCCUCUCUUCUGUCAA | Cleavage |
| ptc-miR156g/h/i/j | SPL6/XM_011013358.1 | 2 | 1 | 21 | 1195 | 1215 | UUGACAGAAGAUAGAGAGCAC | GUGCUCUCUCUCUUCUGUCAG | Cleavage |
| ptc-miR156g/h/i/j | SPL6/XM_011020626.1 | 2 | 1 | 21 | 1280 | 1300 | UUGACAGAAGAUAGAGAGCAC | GUGCUCUCUCUCUUCUGUCAG | Cleavage |
| ptc-miR156g/h/i/j | SPL7/XM_011019136.1 | 2 | 1 | 21 | 1130 | 1150 | UUGACAGAAGAUAGAGAGCAC | GUGCUCUCUCUCUUCUGUCAU | Cleavage |
| ptc-miR156g/h/i/j | SPL7/XM_011044186.1 | 2 | 1 | 21 | 833 | 853 | UUGACAGAAGAUAGAGAGCAC | GUGCUCUCUCUCUUCUGUCAU | Cleavage |
| ptc-miR156g/h/i/j | SPL9/XM_011034944.1 | 1 | 1 | 21 | 1095 | 1115 | UUGACAGAAGAUAGAGAGCAC | GUGCUCUCUCUCUUCUGUCAA | Cleavage |
| ptc-miR156k | SPL10/XM_011016285.1 | 1 | 1 | 20 | 1857 | 1876 | UGACAGAAGAGAGGGAGCAC | GUGCUCUCUCUCUUCUGUCA | Cleavage |
| ptc-miR156k | SPL10/XM_011016288.1 | 1 | 1 | 20 | 1836 | 1855 | UGACAGAAGAGAGGGAGCAC | GUGCUCUCUCUCUUCUGUCA | Cleavage |
| ptc-miR156k | SPL10/XM_011016290.1 | 1 | 1 | 20 | 1806 | 1825 | UGACAGAAGAGAGGGAGCAC | GUGCUCUCUCUCUUCUGUCA | Cleavage |
| ptc-miR156k | SPL10/XM_011016291.1 | 1 | 1 | 20 | 1785 | 1804 | UGACAGAAGAGAGGGAGCAC | GUGCUCUCUCUCUUCUGUCA | Cleavage |
| ptc-miR156k | SPL13A/XM_011025667.1 | 1 | 1 | 20 | 1638 | 1657 | UGACAGAAGAGAGGGAGCAC | GUGCUCUCUCUCUUCUGUCA | Cleavage |
| ptc-miR156k | SPL13B/XM_011022482.1 | 1 | 1 | 20 | 1380 | 1399 | UGACAGAAGAGAGGGAGCAC | GUGCUCUCUCUCUUCUGUCA | Cleavage |
| ptc-miR156k | SPL13B/XM_011037799.1 | 1 | 1 | 20 | 1469 | 1488 | UGACAGAAGAGAGGGAGCAC | GUGCUCUCUCUCUUCUGUCA | Cleavage |
| ptc-miR156k | SPL2/XM_011003831.1 | 1 | 1 | 20 | 2028 | 2047 | UGACAGAAGAGAGGGAGCAC | GUGCUCUCUCUCUUCUGUCA | Cleavage |
| ptc-miR156k | SPL2/XM_011022433.1 | 1 | 1 | 20 | 1970 | 1989 | UGACAGAAGAGAGGGAGCAC | GUGCUCUCUCUCUUCUGUCA | Cleavage |
| ptc-miR156k | SPL2/XM_011022434.1 | 1 | 1 | 20 | 1960 | 1979 | UGACAGAAGAGAGGGAGCAC | GUGCUCUCUCUCUUCUGUCA | Cleavage |
| ptc-miR156k | SPL2/XM_011049364.1 | 2 | 1 | 20 | 879 | 898 | UGACAGAAGAGAGGGAGCAC | UUGCUCUCUCUCUUCUGUCA | Cleavage |
| ptc-miR156k | SPL3/XM_011029495.1 | 2 | 1 | 20 | 721 | 740 | UGACAGAAGAGAGGGAGCAC | AUGCUCUCUCUCUUCUGUCA | Cleavage |
| ptc-miR156k | SPL3/XM_011029496.1 | 2 | 1 | 20 | 701 | 720 | UGACAGAAGAGAGGGAGCAC | AUGCUCUCUCUCUUCUGUCA | Cleavage |
| ptc-miR156k | SPL3/XM_011045108.1 | 1 | 1 | 20 | 726 | 745 | UGACAGAAGAGAGGGAGCAC | AUGCUCUCUCUCUUCUGUCA | Cleavage |
| ptc-miR156k | SPL4/XM_011031202.1 | 1 | 1 | 20 | 894 | 913 | UGACAGAAGAGAGGGAGCAC | AUGCUCCCUCUCUUCUGUCA | Cleavage |
| ptc-miR156k | SPL6/XM_011013358.1 | 1 | 1 | 20 | 1195 | 1214 | UGACAGAAGAGAGGGAGCAC | GUGCUCUCUCUCUUCUGUCA | Cleavage |
| ptc-miR156k | SPL6/XM_011020626.1 | 1 | 1 | 20 | 1280 | 1299 | UGACAGAAGAGAGGGAGCAC | GUGCUCUCUCUCUUCUGUCA | Cleavage |
| ptc-miR156k | SPL7/XM_011019136.1 | 1 | 1 | 20 | 1130 | 1149 | UGACAGAAGAGAGGGAGCAC | GUGCUCUCUCUCUUCUGUCA | Cleavage |
| ptc-miR156k | SPL7/XM_011044186.1 | 1 | 1 | 20 | 833 | 852 | UGACAGAAGAGAGGGAGCAC | GUGCUCUCUCUCUUCUGUCA | Cleavage |
| ptc-miR156k | SPL9/XM_011034944.1 | 1 | 1 | 20 | 1095 | 1114 | UGACAGAAGAGAGGGAGCAC | GUGCUCUCUCUCUUCUGUCA | Cleavage |
| ptc-miR156l | SPL10/XM_011016285.1 | 2 | 1 | 21 | 1857 | 1877 | UUGACAGAAGAUGGAGAGCAC | GUGCUCUCUCUCUUCUGUCAA | Cleavage |
| ptc-miR156l | SPL10/XM_011016288.1 | 2 | 1 | 21 | 1836 | 1856 | UUGACAGAAGAUGGAGAGCAC | GUGCUCUCUCUCUUCUGUCAA | Cleavage |
| ptc-miR156l | SPL10/XM_011016290.1 | 2 | 1 | 21 | 1806 | 1826 | UUGACAGAAGAUGGAGAGCAC | GUGCUCUCUCUCUUCUGUCAA | Cleavage |
| ptc-miR156l | SPL10/XM_011016291.1 | 2 | 1 | 21 | 1785 | 1805 | UUGACAGAAGAUGGAGAGCAC | GUGCUCUCUCUCUUCUGUCAA | Cleavage |
| ptc-miR156l | SPL13A/XM_011025667.1 | 3 | 1 | 21 | 1638 | 1658 | UUGACAGAAGAUGGAGAGCAC | GUGCUCUCUCUCUUCUGUCAU | Cleavage |
| ptc-miR156l | SPL13B/XM_011022482.1 | 3 | 1 | 21 | 1380 | 1400 | UUGACAGAAGAUGGAGAGCAC | GUGCUCUCUCUCUUCUGUCAU | Cleavage |
| ptc-miR156l | SPL13B/XM_011037799.1 | 3 | 1 | 21 | 1469 | 1489 | UUGACAGAAGAUGGAGAGCAC | GUGCUCUCUCUCUUCUGUCAU | Cleavage |
| ptc-miR156l | SPL2/XM_011003831.1 | 2 | 1 | 21 | 2028 | 2048 | UUGACAGAAGAUGGAGAGCAC | GUGCUCUCUCUCUUCUGUCAA | Cleavage |
| ptc-miR156l | SPL2/XM_011022433.1 | 2 | 1 | 21 | 1970 | 1990 | UUGACAGAAGAUGGAGAGCAC | GUGCUCUCUCUCUUCUGUCAA | Cleavage |
| ptc-miR156l | SPL2/XM_011022434.1 | 2 | 1 | 21 | 1960 | 1980 | UUGACAGAAGAUGGAGAGCAC | GUGCUCUCUCUCUUCUGUCAA | Cleavage |
| ptc-miR156l | SPL2/XM_011049364.1 | 3 | 1 | 20 | 880 | 899 | UUGACAGAAGAUGGAGAGCA | UGCUCUCUCUCUUCUGUCAU | Cleavage |
| ptc-miR156l | SPL3/XM_011029495.1 | 2 | 1 | 20 | 722 | 741 | UUGACAGAAGAUGGAGAGCA | UGCUCUCUCUCUUCUGUCAA | Cleavage |
| ptc-miR156l | SPL3/XM_011029496.1 | 2 | 1 | 20 | 702 | 721 | UUGACAGAAGAUGGAGAGCA | UGCUCUCUCUCUUCUGUCAA | Cleavage |
| ptc-miR156l | SPL3/XM_011045108.1 | 2 | 1 | 20 | 727 | 746 | UUGACAGAAGAUGGAGAGCA | UGCUCUCUCUCUUCUGUCAA | Cleavage |
| ptc-miR156l | SPL4/XM_011031202.1 | 3 | 1 | 20 | 895 | 914 | UUGACAGAAGAUGGAGAGCA | UGCUCCCUCUCUUCUGUCAA | Cleavage |
| ptc-miR156l | SPL6/XM_011013358.1 | 2 | 1 | 21 | 1195 | 1215 | UUGACAGAAGAUGGAGAGCAC | GUGCUCUCUCUCUUCUGUCAG | Cleavage |
| ptc-miR156l | SPL6/XM_011020626.1 | 2 | 1 | 21 | 1280 | 1300 | UUGACAGAAGAUGGAGAGCAC | GUGCUCUCUCUCUUCUGUCAG | Cleavage |
| ptc-miR156l | SPL7/XM_011019136.1 | 3 | 1 | 21 | 1130 | 1150 | UUGACAGAAGAUGGAGAGCAC | GUGCUCUCUCUCUUCUGUCAU | Cleavage |
| ptc-miR156l | SPL7/XM_011044186.1 | 3 | 1 | 21 | 833 | 853 | UUGACAGAAGAUGGAGAGCAC | GUGCUCUCUCUCUUCUGUCAU | Cleavage |
| ptc-miR156l | SPL9/XM_011034944.1 | 2 | 1 | 21 | 1095 | 1115 | UUGACAGAAGAUGGAGAGCAC | GUGCUCUCUCUCUUCUGUCAA | Cleavage |
| ptc-miR156a/b/c/d/e/f | TCONS_00004524 | 3 | 1 | 20 | 930 | 949 | UGACAGAAGAGAGUGAGCAC | UUUCCCAUUUUCUUCUGUCA | Cleavage |
| ptc-miR156a/b/c/d/e/f | XR_842262.1 | 5 | 1 | 20 | 93 | 112 | UGACAGAAGAGAGUGAGCAC | GGGUUCUCUCUUUUUUGUCU | Cleavage |
| ptc-miR156a/b/c/d/e/f | XR_842376.1 | 4 | 1 | 20 | 135 | 154 | UGACAGAAGAGAGUGAGCAC | UUGUUGUCAUUCUUCUGUCA | Cleavage |
| ptc-miR156a/b/c/d/e/f | XR_842556.1 | 5 | 1 | 20 | 811 | 830 | UGACAGAAGAGAGUGAGCAC | GUUCUCUUUUCUUUCUGUCA | Translation |
| ptc-miR156a/b/c/d/e/f | XR_843316.1 | 5 | 1 | 20 | 73 | 92 | UGACAGAAGAGAGUGAGCAC | GUUCUUGUUCUAUUUUGUUG | Translation |
| ptc-miR156g/h/i/j | TCONS_00039386 | 5 | 1 | 21 | 269 | 289 | UUGACAGAAGAUAGAGAGCAC | GUCUUCUUUAUCUAUAGUCAA | Cleavage |
| ptc-miR156g/h/i/j | XR_839864.1 | 5 | 1 | 21 | 3236 | 3256 | UUGACAGAAGAUAGAGAGCAC | AUGUUUUAUAUAUUGUGUCAG | Translation |
| ptc-miR156g/h/i/j | XR_841995.1 | 5 | 1 | 21 | 266 | 286 | UUGACAGAAGAUAGAGAGCAC | UUUCUUUCUUUCUUUUUUUAA | Cleavage |
| ptc-miR156g/h/i/j | XR_842262.1 | 5 | 1 | 21 | 93 | 113 | UUGACAGAAGAUAGAGAGCAC | GGGUUCUCUCUUUUUUGUCUU | Cleavage |
| ptc-miR156g/h/i/j | XR_842376.1 | 5 | 1 | 21 | 135 | 155 | UUGACAGAAGAUAGAGAGCAC | UUGUUGUCAUUCUUCUGUCAC | Cleavage |
| ptc-miR156g/h/i/j | XR_842556.1 | 5 | 1 | 21 | 811 | 831 | UUGACAGAAGAUAGAGAGCAC | GUUCUCUUUUCUUUCUGUCAC | Translation |
| ptc-miR156g/h/i/j | XR_842721.1 | 4 | 1 | 21 | 479 | 499 | UUGACAGAAGAUAGAGAGCAC | AUGUUCUUUACCUUAUGUUAA | Translation |
| ptc-miR156g/h/i/j | XR_843669.1 | 5 | 1 | 21 | 1676 | 1696 | UUGACAGAAGAUAGAGAGCAC | UUGCUCGCUGUUUAGUGUCAA | Cleavage |
| ptc-miR156g/h/i/j | XR_843841.1 | 5 | 1 | 21 | 594 | 614 | UUGACAGAAGAUAGAGAGCAC | GUUUUUUUUUUUUUUUGUUAA | Cleavage |
| ptc-miR156g/h/i/j | XR_843857.1 | 5 | 1 | 21 | 142 | 162 | UUGACAGAAGAUAGAGAGCAC | CCCUUUUCGAUUUUUUGUUAA | Cleavage |
| ptc-miR156g/h/i/j | XR_844845.1 | 5 | 1 | 21 | 654 | 674 | UUGACAGAAGAUAGAGAGCAC | UGAUUCUCUUUCUUCUGUUCA | Cleavage |
| ptc-miR156g/h/i/j | XR_845290.1 | 5 | 1 | 21 | 59 | 79 | UUGACAGAAGAUAGAGAGCAC | UUGUUUUACAUCUUUUUUCAA | Cleavage |
| ptc-miR156g/h/i/j | XR_845290.1 | 5 | 1 | 21 | 1447 | 1467 | UUGACAGAAGAUAGAGAGCAC | UCGCUUUUUGUUUCUUGUGAA | Cleavage |
| ptc-miR156g/h/i/j | XR_845292.1 | 5 | 1 | 21 | 51 | 71 | UUGACAGAAGAUAGAGAGCAC | UUGUUUUACAUCUUUUUUCAA | Cleavage |
| ptc-miR156g/h/i/j | XR_845292.1 | 5 | 1 | 21 | 1439 | 1459 | UUGACAGAAGAUAGAGAGCAC | UCGCUUUUUGUUUCUUGUGAA | Cleavage |
| ptc-miR156g/h/i/j | XR_845293.1 | 5 | 1 | 21 | 53 | 73 | UUGACAGAAGAUAGAGAGCAC | UUGUUUUACAUCUUUUUUCAA | Cleavage |
| ptc-miR156g/h/i/j | XR_845293.1 | 5 | 1 | 21 | 1441 | 1461 | UUGACAGAAGAUAGAGAGCAC | UCGCUUUUUGUUUCUUGUGAA | Cleavage |
| ptc-miR156k | TCONS_00028715 | 5 | 1 | 20 | 210 | 229 | UGACAGAAGAGAGGGAGCAC | GUGUUUCCUUUCCUUUAUCA | Cleavage |
| ptc-miR156k | TCONS_00028715 | 5 | 1 | 20 | 1611 | 1630 | UGACAGAAGAGAGGGAGCAC | AUGUGCCCUCCCUUUUUUUA | Translation |
| ptc-miR156k | TCONS_00066459 | 5 | 1 | 20 | 143 | 162 | UGACAGAAGAGAGGGAGCAC | GCACUCCUUCAUUUCUGUCG | Translation |
| ptc-miR156k | TCONS_00069472 | 5 | 1 | 20 | 422 | 441 | UGACAGAAGAGAGGGAGCAC | CAUUUACCUCUCUUCUGUUU | Cleavage |
| ptc-miR156k | TCONS_00070591 | 5 | 1 | 20 | 21 | 40 | UGACAGAAGAGAGGGAGCAC | GUUUUCCUUUUCUGCUGUCU | Cleavage |
| ptc-miR156k | XR_840123.1 | 5 | 1 | 20 | 946 | 965 | UGACAGAAGAGAGGGAGCAC | GUGCUACUUUGCUUCGGUCA | Translation |
| ptc-miR156k | XR_841846.1 | 5 | 1 | 20 | 464 | 483 | UGACAGAAGAGAGGGAGCAC | GUCCUCCUUCCUUUCUCUUA | Translation |
| ptc-miR156k | XR_842262.1 | 4 | 1 | 20 | 93 | 112 | UGACAGAAGAGAGGGAGCAC | GGGUUCUCUCUUUUUUGUCU | Cleavage |
| ptc-miR156k | XR_842376.1 | 4 | 1 | 20 | 135 | 154 | UGACAGAAGAGAGGGAGCAC | UUGUUGUCAUUCUUCUGUCA | Cleavage |
| ptc-miR156k | XR_842556.1 | 4 | 1 | 20 | 811 | 830 | UGACAGAAGAGAGGGAGCAC | GUUCUCUUUUCUUUCUGUCA | Translation |
| ptc-miR156k | XR_842721.1 | 5 | 1 | 21 | 479 | 499 | UUGACAGAAGAUGGAGAGCAC | AUGUUCUUUACCUUAUGUUAA | Translation |
| ptc-miR156k | XR_843262.1 | 5 | 1 | 20 | 1973 | 1992 | UGACAGAAGAGAGGGAGCAC | GUGCUCCUUUUUUUUUUUUU | Cleavage |
| ptc-miR156k | XR_843262.1 | 5 | 1 | 21 | 997 | 1017 | UUGACAGAAGAUGGAGAGCAC | UUUCUUUCCAUUUUUUGGUAA | Cleavage |
| ptc-miR156k | XR_843841.1 | 5 | 1 | 20 | 594 | 613 | UGACAGAAGAGAGGGAGCAC | GUUUUUUUUUUUUUUUGUUA | Cleavage |
| ptc-miR156k | XR_844467.1 | 5 | 1 | 20 | 404 | 423 | UGACAGAAGAGAGGGAGCAC | GUGCUGCCUCUUUUCUUUUC | Cleavage |
| ptc-miR156k | XR_844845.1 | 5 | 1 | 20 | 654 | 673 | UGACAGAAGAGAGGGAGCAC | UGAUUCUCUUUCUUCUGUUC | Cleavage |
| ptc-miR156k | XR_845292.1 | 4 | 1 | 21 | 51 | 71 | UUGACAGAAGAUGGAGAGCAC | UUGUUUUACAUCUUUUUUCAA | Cleavage |
| ptc-miR156l | TCONS_00069472 | 5 | 1 | 21 | 488 | 508 | UUGACAGAAGAUGGAGAGCAC | AUGGUCUUCAAUUUUCGUCAA | Translation |
| ptc-miR156l | XR_839697.1 | 5 | 1 | 21 | 433 | 453 | UUGACAGAAGAUGGAGAGCAC | CCACUCUUCAACUUCUGCUAA | Translation |
| ptc-miR156l | XR_839867.1 | 5 | 1 | 21 | 455 | 475 | UUGACAGAAGAUGGAGAGCAC | UUGUUCUCCAGUUCUUGUCAC | Translation |
| ptc-miR156l | XR_839867.1 | 5 | 1 | 21 | 3222 | 3242 | UUGACAGAAGAUGGAGAGCAC | CUUCUCUCCAAUUUUUGACGA | Translation |
| ptc-miR156l | XR_839868.1 | 5 | 1 | 21 | 455 | 475 | UUGACAGAAGAUGGAGAGCAC | UUGUUCUCCAGUUCUUGUCAC | Translation |
| ptc-miR156l | XR_839868.1 | 5 | 1 | 21 | 3222 | 3242 | UUGACAGAAGAUGGAGAGCAC | CUUCUCUCCAAUUUUUGACGA | Translation |
| ptc-miR156l | XR_842144.1 | 5 | 1 | 21 | 2712 | 2732 | UUGACAGAAGAUGGAGAGCAC | GUGUAUUUCAUCUUCUUUCGG | Cleavage |
| ptc-miR156l | XR_842146.1 | 5 | 1 | 21 | 2792 | 2812 | UUGACAGAAGAUGGAGAGCAC | GUGUAUUUCAUCUUCUUUCGG | Cleavage |
| ptc-miR156l | XR_842376.1 | 5 | 1 | 21 | 135 | 155 | UUGACAGAAGAUGGAGAGCAC | UUGUUGUCAUUCUUCUGUCAC | Cleavage |
| ptc-miR156l | XR_843115.1 | 5 | 1 | 21 | 170 | 190 | UUGACAGAAGAUGGAGAGCAC | UUUUUCUUCGUCUUUUGCCAA | Cleavage |
| ptc-miR156l | XR_843669.1 | 5 | 1 | 21 | 1676 | 1696 | UUGACAGAAGAUGGAGAGCAC | UUGCUCGCUGUUUAGUGUCAA | Cleavage |
| ptc-miR156l | XR_843857.1 | 5 | 1 | 21 | 142 | 162 | UUGACAGAAGAUGGAGAGCAC | CCCUUUUCGAUUUUUUGUUAA | Cleavage |
| ptc-miR156l | XR_845290.1 | 4 | 1 | 21 | 59 | 79 | UUGACAGAAGAUGGAGAGCAC | UUGUUUUACAUCUUUUUUCAA | Cleavage |
| ptc-miR156l | XR_845293.1 | 4 | 1 | 21 | 53 | 73 | UUGACAGAAGAUGGAGAGCAC | UUGUUUUACAUCUUUUUUCAA | Cleavage |
| ptc-miR156a/b/c/d/e/f | circRNA_0168 | 5 | 1 | 20 | 9414 | 9433 | UGACAGAAGAGAGUGAGCAC | GGGCUUUUUUUCUUUUGUUG | Cleavage |
| ptc-miR156a/b/c/d/e/f | circRNA_0227 | 5 | 1 | 20 | 10206 | 10225 | UGACAGAAGAGAGUGAGCAC | CUGUUUGGUUUCUUUUGUUU | Cleavage |
| ptc-miR156a/b/c/d/e/f | circRNA_0227 | 5 | 1 | 20 | 5377 | 5396 | UGACAGAAGAGAGUGAGCAC | CUGCUGAAGCUCUUCUGCUA | Cleavage |
| ptc-miR156a/b/c/d/e/f | circRNA_0771 | 5 | 1 | 20 | 8220 | 8239 | UGACAGAAGAGAGUGAGCAC | AUGUUAAUUUUUUUUUAUCA | Cleavage |
| ptc-miR156a/b/c/d/e/f | circRNA_0919 | 5 | 1 | 20 | 35083 | 35102 | UGACAGAAGAGAGUGAGCAC | CCCCGUACUUUUUUCUGUCA | Cleavage |
| ptc-miR156a/b/c/d/e/f | circRNA_0974 | 4 | 1 | 20 | 38252 | 38271 | UGACAGAAGAGAGUGAGCAC | AUCUUCCCUUUUUUUUGUCA | Cleavage |
| ptc-miR156a/b/c/d/e/f | circRNA_0974 | 5 | 1 | 20 | 16289 | 16308 | UGACAGAAGAGAGUGAGCAC | GACCUCAUUUUUUUUUGUUA | Cleavage |
| ptc-miR156a/b/c/d/e/f | circRNA_0974 | 5 | 1 | 20 | 6103 | 6122 | UGACAGAAGAGAGUGAGCAC | AUUUUUAUUAUUUUUUGUUA | Translation |
| ptc-miR156a/b/c/d/e/f | circRNA_1102 | 5 | 1 | 20 | 13232 | 13251 | UGACAGAAGAGAGUGAGCAC | GUGUUCUUUGUCUUUUGUAA | Translation |
| ptc-miR156a/b/c/d/e/f | circRNA_1102 | 5 | 1 | 20 | 1319 | 1338 | UGACAGAAGAGAGUGAGCAC | UUUUUUACUUGAUUUUGUCA | Translation |
| ptc-miR156g/h/i/j | circRNA_0168 | 5 | 1 | 21 | 9414 | 9434 | UUGACAGAAGAUAGAGAGCAC | GGGCUUUUUUUCUUUUGUUGC | Cleavage |
| ptc-miR156g/h/i/j | circRNA_0168 | 5 | 1 | 21 | 1885 | 1905 | UUGACAGAAGAUAGAGAGCAC | GGACUUUCGGUCUUUUCUCAA | Cleavage |
| ptc-miR156g/h/i/j | circRNA_0227 | 4 | 1 | 21 | 6751 | 6771 | UUGACAGAAGAUAGAGAGCAC | AAGUUUUCUAGUUUCUUUCAA | Translation |
| ptc-miR156g/h/i/j | circRNA_0771 | 5 | 1 | 21 | 3464 | 3484 | UUGACAGAAGAUAGAGAGCAC | AACCUUUACAUCUUUUGUCAC | Cleavage |
| ptc-miR156g/h/i/j | circRNA_0826 | 5 | 1 | 21 | 3516 | 3536 | UUGACAGAAGAUAGAGAGCAC | UCCUUUUUUAUUUUUUUUCAA | Cleavage |
| ptc-miR156g/h/i/j | circRNA_0974 | 5 | 1 | 21 | 38252 | 38272 | UUGACAGAAGAUAGAGAGCAC | AUCUUCCCUUUUUUUUGUCAA | Cleavage |
| ptc-miR156g/h/i/j | circRNA_0974 | 5 | 1 | 21 | 36144 | 36164 | UUGACAGAAGAUAGAGAGCAC | UUGGUCUAUAUUUUAUGUCAU | Cleavage |
| ptc-miR156g/h/i/j | circRNA_1102 | 4 | 1 | 21 | 13232 | 13252 | UUGACAGAAGAUAGAGAGCAC | GUGUUCUUUGUCUUUUGUAAA | Cleavage |
| ptc-miR156g/h/i/j | circRNA_1102 | 5 | 1 | 21 | 14933 | 14953 | UUGACAGAAGAUAGAGAGCAC | UAGUUUUUUAUUUUCUUUCUA | Cleavage |
| ptc-miR156k | circRNA_0168 | 5 | 1 | 20 | 9414 | 9433 | UGACAGAAGAGAGGGAGCAC | GGGCUUUUUUUCUUUUGUUG | Cleavage |
| ptc-miR156k | circRNA_0168 | 5 | 1 | 20 | 855 | 874 | UGACAGAAGAGAGGGAGCAC | UUGUUUCUGUUCUUUUAUCA | Cleavage |
| ptc-miR156k | circRNA_0229 | 5 | 1 | 20 | 5120 | 5139 | UGACAGAAGAGAGGGAGCAC | UAGCUACUUUUCUUUUCUCA | Cleavage |
| ptc-miR156k | circRNA_0801 | 5 | 1 | 20 | 1178 | 1197 | UGACAGAAGAGAGGGAGCAC | GUCCUCUUGCUCUUCUCUCG | Cleavage |
| ptc-miR156k | circRNA_0882 | 5 | 1 | 20 | 1178 | 1197 | UGACAGAAGAGAGGGAGCAC | GUCCUCUUGCUCUUCUCUCG | Cleavage |
| ptc-miR156k | circRNA_0974 | 3 | 1 | 20 | 38252 | 38271 | UGACAGAAGAGAGGGAGCAC | AUCUUCCCUUUUUUUUGUCA | Cleavage |
| ptc-miR156k | circRNA_0974 | 5 | 1 | 20 | 12532 | 12551 | UGACAGAAGAGAGGGAGCAC | CUCUUCCCUUUCUUCUUUUU | Cleavage |
| ptc-miR156k | circRNA_0974 | 5 | 1 | 20 | 25818 | 25837 | UGACAGAAGAGAGGGAGCAC | UUCUUUUUUUUCUUCGGUCA | Cleavage |
| ptc-miR156k | circRNA_1102 | 5 | 1 | 20 | 13232 | 13251 | UGACAGAAGAGAGGGAGCAC | GUGUUCUUUGUCUUUUGUAA | Translation |
| ptc-miR156l | circRNA_0168 | 5 | 1 | 21 | 9414 | 9434 | UUGACAGAAGAUGGAGAGCAC | GGGCUUUUUUUCUUUUGUUGC | Cleavage |
| ptc-miR156l | circRNA_0168 | 5 | 1 | 21 | 1885 | 1905 | UUGACAGAAGAUGGAGAGCAC | GGACUUUCGGUCUUUUCUCAA | Cleavage |
| ptc-miR156l | circRNA_0227 | 5 | 1 | 21 | 6751 | 6771 | UUGACAGAAGAUGGAGAGCAC | AAGUUUUCUAGUUUCUUUCAA | Translation |
| ptc-miR156l | circRNA_0227 | 5 | 1 | 21 | 5000 | 5020 | UUGACAGAAGAUGGAGAGCAC | GAACUCUCCAUUUUUUGUUUU | Cleavage |
| ptc-miR156l | circRNA_0840 | 5 | 1 | 21 | 11453 | 11473 | UUGACAGAAGAUGGAGAGCAC | UCAACCGUCAUUUUCUGUCAA | Cleavage |
| ptc-miR156l | circRNA_0950 | 4 | 1 | 21 | 9049 | 9069 | UUGACAGAAGAUGGAGAGCAC | CAGCUGUCCAUCCUCUGUCUA | Translation |
| ptc-miR156l | circRNA_0974 | 5 | 1 | 21 | 3262 | 3282 | UUGACAGAAGAUGGAGAGCAC | GUGAUCUUCAUCCUCUGUAAU | Translation |
| ptc-miR156l | circRNA_0974 | 5 | 1 | 21 | 3774 | 3794 | UUGACAGAAGAUGGAGAGCAC | ACUCUCUUCAUUUUCUAGCAA | Cleavage |
| ptc-miR156l | circRNA_0979 | 4 | 1 | 21 | 4505 | 4525 | UUGACAGAAGAUGGAGAGCAC | UGGCUCUUCAUCCUCUUUUAG | Translation |
| ptc-miR156l | circRNA_1102 | 4 | 1 | 21 | 13232 | 13252 | UUGACAGAAGAUGGAGAGCAC | GUGUUCUUUGUCUUUUGUAAA | Cleavage |
