# Supplementary material for: Identification and regulatory network analysis of SPL family transcription factors in Populus euphratica Oliv. heteromorphic leaves
Source: Sci Rep. 2022 Feb 21;12:2856. doi: 10.1038/s41598-022-06942-w (PMC8861001; doi:10.1038/s41598-022-06942-w)
Supplement: Supplementary file 2 — Supplementary Figure S2. [file 41598_2022_6942_MOESM2_ESM.pdf]

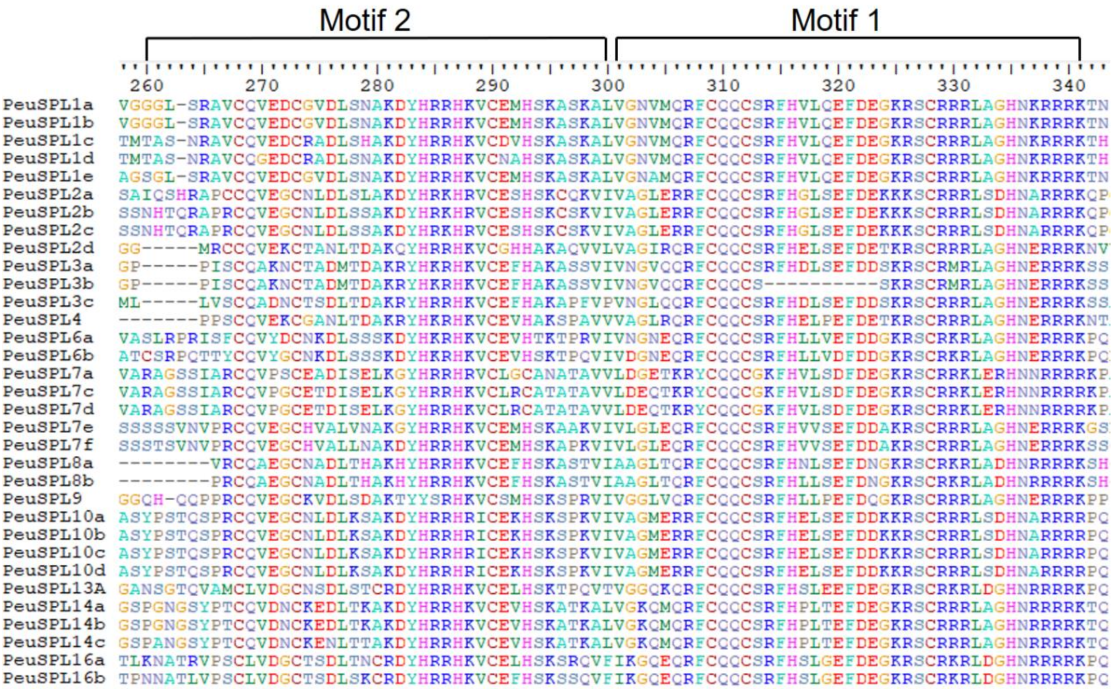

Figure S2. Multiple sequence alignment and of motif 1 and motif 2. This figure was generated by COBALT (https://www.ncbi.nlm.nih.gov/tools/cobalt/).
